# Correction: In Vitro and In Vivo Metabolism and Inhibitory Activities of Vasicine, a Potent Acetylcholinesterase and Butyrylcholinesterase Inhibitor

**DOI:** 10.1371/journal.pone.0129759

**Published:** 2015-06-05

**Authors:** The PLOS ONE Staff

## Notice of Republication

This article was republished on May 6, 2015, to correct an error which occurred only in the PDF. The first two sentences of the second paragraph under the subheading "Metabolites M3" under the heading "Characterization of the metabolites in vivo and in vitro" in the Materials and Methods section were not correctly included in the original PDF.

The publisher apologizes for the errors. Please download this article again to view the correct version. The originally published, uncorrected article and the republished, corrected article are provided here for reference.

## Supporting Information

S1 File Originally published, uncorrected article. (PDF)

S2 File Republished corrected article. (PDF)
