# Supplementary material for: Correction: In Vitro and In Vivo Metabolism and Inhibitory Activities of Vasicine, a Potent Acetylcholinesterase and Butyrylcholinesterase Inhibitor
Source: PLoS One. 2015 Jun 5;10(6):e0129759. doi: 10.1371/journal.pone.0129759 (PMC4457867; doi:10.1371/journal.pone.0129759)
Supplement: S2 File — (PDF) [file pone.0129759.s002.pdf]

RESEARCH ARTICLE

In Vitro and In Vivo Metabolism and Inhibitory Activities of Vasicine, a Potent Acetylcholinesterase and Butyrylcholinesterase Inhibitor

Wei Liu[1], Xiaoyuan Shi[1], Yadi Yang[1], Xuemei Cheng[3], Qing Liu[3], Han Han[1], Baohua Yang[1], Chunyong He[1], Yongli Wang[1,3], Bo Jiang[1], Zhengtao Wang[1,2,3], Changhong Wang[1,2,3]*

1 Institute of Chinese Materia Medica, Shanghai University of Traditional Chinese Medicine, Shanghai, China, **2** The MOE Key Laboratory for Standardization of Chinese Medicines and The SATCM Key Laboratory for New Resources and Quality Evaluation of Chinese Medicines, Shanghai, China, **3** Shanghai R&D Centre for Standardization of Chinese Medicines, Shanghai, China

* wchcxm@hotmail.com

OPEN ACCESS

Citation: Liu W, Shi X, Yang Y, Cheng X, Liu Q, Han H, et al. (2015) In Vitro and In Vivo Metabolism and Inhibitory Activities of Vasicine, a Potent Acetylcholinesterase and Butyrylcholinesterase Inhibitor. PLoS ONE 10(4): e0122366. doi:10.1371/journal.pone.0122366

Academic Editor: Israel Silman, Weizmann Institute of Science, ISRAEL

Received: July 30, 2014

Accepted: February 11, 2015

Published: April 7, 2015

Copyright: © 2015 Liu et al. This is an open access article distributed under the terms of the Creative Commons Attribution License, which permits unrestricted use, distribution, and reproduction in any medium, provided the original author and source are credited.

Data Availability Statement: All relevant data are within the paper.

Funding: This work was financially supported by the Key Projects of Joint Funds of the National Natural Science Foundation of China and Xinjiang Uygur Autonomous Region of China (No. U1130303), the National Natural Science Foundation of China (Grant 81173119), the National Science & Technology Major Project "Key New Drug Creation and Manufacturing Program", China (Grants 2012ZX0910320-051), and the Program of Shanghai Subject Chief Scientist (13XD1403500) awarded to Professor Chang-hong

Abstract

Vasicine (VAS), a potential natural cholinesterase inhibitor, exhibited promising anticholinesterase activity in preclinical models and has been in development for treatment of Alzheimer's disease. This study systematically investigated the in vitro and in vivo metabolism of VAS in rat using ultra performance liquid chromatography combined with electrospray ionization quadrupole time-of-flight mass spectrometry. A total of 72 metabolites were found based on a detailed analysis of their ^{1}H- NMR and ^{13}C NMR data. Six key metabolites were isolated from rat urine and elucidated as vasicinone, vasicinol, vasicinolone, 1,2,3,9-tetrahydropyrrolo [2,1-b] quinazolin-3-yl hydrogen sulfate, 9-oxo-1,2,3,9-tetrahydropyrrolo [2,1-b] quinazolin-3-yl hydrogen sulfate, and 1,2,3,9-tetrahydropyrrolo [2,1-b] quinazolin-3-β-D-glucuronide. The metabolic pathway of VAS in vivo and in vitro mainly involved monohydroxylation, dihydroxylation, trihydroxylation, oxidation, desaturation, sulfation, and glucuronidation. The main metabolic soft spots in the chemical structure of VAS were the 3-hydroxyl group and the C-9 site. All 72 metabolites were found in the urine sample, and 15, 25, 45, 18, and 11 metabolites were identified from rat feces, plasma, bile, rat liver microsomes, and rat primary hepatocyte incubations, respectively. Results indicated that renal clearance was the major excretion pathway of VAS. The acetylcholinesterase (AChE) and butyrylcholinesterase (BChE) inhibitory activities of VAS and its main metabolites were also evaluated. The results indicated that although most metabolites maintained potential inhibitory activity against AChE and BChE, but weaker than that of VAS. VAS undergoes metabolic inactivation process in vivo in respect to cholinesterase inhibitory activity.

Wang for financial support of this study. The funders had no role in study design, data collection and analysis, decision to publish, or preparation of the manuscript.

Competing Interests: The authors have declared that no competing interests exist.

Introduction

Vasicine (VAS), a pyrrolo [2,1-b] quinazoline type alkaloid, is the main active component in *Adhatoda vasica* Nees (*Acanthaceae*) and *Peganum harmala* Linn (*Zygophyllaceae*) [1–3]. *A. vasica* is a popular Indian medicinal plant that has been used in Ayurvedic medicine system for treatment of various ailments of the respiratory tract in both children and adults [4]. *P. harmala* is also an effective herbal medicine that has been widely used to treat various diseases, such as hypertension, diabetes, asthma, and rheumatism in China and the Middle East [5, 6]. Moreover, *P. harmala* has been mentioned in our previous studies, wherein we reported that VAS has a strong inhibitive activity against acetylcholinesterase (AChE) and butyrylcholinesterase (BChE), with IC_{50} values of 3.24 ± 0.08 μM and 0.10 ± 0.00 μM, respectively [3, 6–8]. It implied that VAS can be used for treatment of Alzheimer's disease. Other pharmacological activities, such as antimicrobial, anti-inflammatory [9], antioxidant [10], bronchodilatory [11], antitussive [12], and abortifacient effects [13] have been reported for VAS.

Metabolic stability and metabolic profile are essential in designing new drugs because the metabolism of drugs can induce many pharmacokinetic (low bioavailability, high clearance, short half-life, first-pass elimination) and toxic problems (reactive metabolites) that can be regulated in the early stages of development by identifying the relationship between metabolism, metabolic toxicity, and compound structure [14, 15]. Previous studies have reported that VAS could be metabolized into vasicinone (VAO), desoxyvasicine (DVAS), deoxyvasicinone (DVAO), 1,2,3,9-tetrahydropyrrolo [2,1-b] quinazolin-3-β-D- glucuronid (VAS-3-G), and vasicinone-3-*O*-glucuronide (VAO-3-G) in rats after an oral dose of 20 mg/kg [16]. However, a discrepancy result was observed in our preliminary experiment, in which DVAS and DVAO were not found among the metabolites.

Characterizing the chemical structure of metabolites is important in metabolism studies [17, 18]. Determining the exact structure of metabolites is also the nodus in metabolism studies. Ultra-performance liquid chromatography combined with electrospray ionization quadrupole time-of-flight tandem mass spectrometry (UPLC/ESI-QTOF-MS) and the chemical intelligent software tool Mass Fragment have been used in the metabolic identification of xenobiotics in recent years [19–23]. In the present study, UPLC/ESI QTOF MS was used to perform in vivo and in vitro metabolite identification studies on VAS in rat to elucidate the real metabolism of VAS and understand its whole metabolic route. The metabolites were easily found by comparing fragmentation ions obtained from MS and chromatographic behaviors of the authentic standards. However, identification and characterization of metabolite structures remain a challenge because of the complicated mass fragmentation patterns in the pyrrolo [2,1-b] quinazoline skeleton of VAS. Moreover, certain metabolites are isomers having the same molecular formula with minimal variations presented in their MS spectra. Therefore, we systematically investigated the fragmentation pathways of pyrrolo [2,1-b] quinazoline alkaloids and found that the mass cleavages of the ring skeleton are characteristics of and related to the chemical structure. Fragmentation patterns can be used as a critical clue in investigating VAS metabolism. As a result, a total of 72 metabolites of VAS have been identified in vivo from rat bio-specimens (urine, feces, plasma, and bile) and in vitro from rat liver microsomes (RLMs) and rat primary hepatocytes (RPHs) incubation.

Materials and Methods

Chemicals and reagents

VAS and VAO were isolated from *P. harmala* in our laboratory according to a previously reported method [24]. VAS and VAO structures were elucidated by comparing their spectral

data (UV, MS, ^{1}H-NMR, and ^{13}C NMR) with the references. The purities of VAS and VAO were determined to be more than 98% through normalization of the peak areas detected by HPLC-DAD, UPLC-MS/MS, ^{1}H-NMR, and ^{13}C NMR. AChE from *Electrophorus electricus*, BChE from equine serum, acetylcholine (ACh) chloride, butyrocholine (BCh) chloride, choline (Ch) chloride, chlormequat (internal standard, IS), glucose 6-phosphate, glucose-6-phosphate dehydrogenase, NADP, UDPGA, alamethicin, fetal bovine serum, dexamethasone, and insulin were purchased from Sigma Aldrich Co. (St. Louis, MO, USA). Tris base and $MgCl_2$ were purchased from Majorbio Biotech Corp., Ltd. (Shanghai, China). MCI Gel CHP 20P was purchased from Mitsubishi Chemical Crop. (Tokyo, Japan). Silica Gel C18 (MB 100-40/75) was purchased from Fuji Silysia Chemical Ltd. (Kasugai, Japan). Acetonitrile and methanol of HPLC grade were purchased from Fisher Scientific Co. (Santa Clara, USA). HPLC grade 96% formic acid was purchased from Tedia Co. (Fairfield, USA). Water was produced with a Milli-Q Academic System (Millipore, Billerica, MA). All other reagents and solvents were either analytical or HPLC grade.

Ethics Statement

Animals were maintained and experiments were conducted in accordance with the Institutional Animal Care and Use Committee, Shanghai University of Traditional Chinese Medicine, and with the recommendations in the Guide for the Care and Use of Laboratory Animals of the National Institutes of Health. The study was approved by the Animal Care and Use Committee of Shanghai University of Traditional Chinese Medicine (Approval Number: ACSHU-2011-G115).

Animals

Male Sprague–Dawley rats (200 g to 250 g) were obtained from the Experimental Animal Center, Shanghai University of Traditional Chinese Medicine (Permit Number: SCXK (Hu) 2013–0016) and housed with free access to food and water. The animals were maintained on a 12/12 h light and dark cycle at room temperature (22°C to 24°C) and relative humidity (60% to 65%) for 7 days. All rats were fasted for 12 h and fed with water before the experiment.

Preparation of RLMs

Rat livers were obtained from corresponding healthy experimental animals from the Experimental Animal Center of Shanghai University of Traditional Chinese Medicine (SUTCM, Shanghai, China). Liver specimens were stored in liquid nitrogen immediately after being harvested until the preparation of microsomes. RLMs were prepared from pooled liver tissues by differential ultracentrifugation, as previously described [25]. Protein concentration was determined using bovine serum albumin as a reference [26]. RLMs were diluted to 10 mg/mL and stored at −80°C.

Drug administration and sample collection

Bio-specimens (urine, feces, bile and plasma) collected form 18 male rats were used to study the VAS metabolism in vivo. Urine and fecal samples of six male rats were collected from 0 to 24 h after oral administration of 50 mg/kg (10 mg/mL) VAS dissolved in water with hydrochloride as pH regulator. Baseline urine and fecal samples were collected before oral administration of VAS. After intraperitoneal injection of urethane (1.4 g/kg), abdominal incision was made for bile sampling and the common bile duct was cannulated with a Closed IV Catheter System (ID = 0.07 cm, Becton Dickinson, USA) to collect bile samples. Bile samples from additional

six male rats were collected from 0 to 12 h after oral administration of 50 mg/kg VAS. Baseline rat bile was collected before oral administration of VAS. All the samples were stored at −20°C until analysis. Blood was collected from the angular vein of the last six male rats at 0.25, 0.5, 1, and 2 h after oral administration of VAS 50 mg/kg VAS. Plasma was obtained by centrifuging blood at 4000 × *g* for 10 min, and then stored at −20°C until use.

The urine samples used for isolating and purifying the metabolites were collected from 15 rats that were given VAS orally once a day, over 21 consecutive days, at dose of 100 mg/kg body weight. In total, approximately 5 L of urine was collected and centrifuged at 4000 × *g* for 15 min. The supernatant was then stored at −20°C until use. At the end of experiment, all rats were sacrificed by CO_2 asphyxiation.

Sample preparation

Up to 1 mL of urine was thoroughly mixed with same volume of acetonitrile, and then centrifuged at 15,000 × *g* for 10 min. The supernatant was evaporated until dry under nitrogen at 37°C. The residue of the supernatant was re-dissolved in 200 μL of 5% methanol, and then centrifuged at 15,000 × *g* for 10 min. The supernatant was injected into the chromatographic systems for separation and identification of metabolites.

Approximately 1 g of rat feces was exhaustively extracted with 40 mL of 75% acetonitrile by ultrasonication at room temperature. The supernatant was then evaporated to dryness by a gentle stream of nitrogen at 37°C. The residue was dissolved by 200 μL of 5% methanol and centrifuged at 15,000 × *g* for 10 min prior to injection.

Plasma or bile (400 μL) was mixed and vortexed with 1200 μL of acetonitrile for 30, then centrifuged at 15,000 × *g* for 10 min at 4°C. Afterward, the supernatant was evaporated until dry by a gentle stream of nitrogen at 37°C. The residue was dissolved by 80 μL of 5% methanol and centrifuged at 15,000 × *g* for 10 min. The supernatants (5 μL) were used in the UPLC/ESI-QTOF-MS analysis.

Isolation and purification of metabolites from urine

Approximately 5 L of urine samples were subjected to MCI Gel CHP 20P column chromatography (4.5 × 60 cm; 1000 mL) and eluted with a gradient system of MeOH—H_2O (0:100; 5:95; 10:90; 20:80; 30:70; 50:50; 100:0). The metabolites were obtained from 0%, 5%, 20%, and 50% MeOH fractions. Each fraction solution was concentrated in vacuo to yield residues. The residues were dissolved in a small amount of water, subjected to a reverse phase silica gel C18 column (1.2 × 30 cm; 15 mL), and then washed with MeOH—H_2O by gradient elution, according to the flowchart for metabolite isolation shown in Fig 1, to afford M5-2 (10 mg), M15 (55 mg), M20-2, M16, and M3-2 (8 mg). The purity of M15, M3-2, and M5-2 were more than 98% as determined normalization of the peak areas detected by HPLC-DAD and UPLC-MS/MS. M16 and M20-2 were further purified by semi-preparative HPLC.

Semi-preparative HPLC was performed with a ZORBAX SB-C18 (9.4 mm × 25 cm, 5 μm, Agilent, USA) at 30°C in a LC3000 liquid chromatography (Beijing Tong Heng Innovation Technology Co., Beijing, China). The detection wavelength was set at 280 nm. The mobile phase consisted of methanol (A) and 0.1% aqueous formic acid (B). For M16, a flow rate of 4.7 mL/min and 18% A isocratic elution were used to obtain M16 (32 mg). M20-2 (20 mg) was obtained using gradient elution: 0 to 10 min, 5% to 20% A, with a flow rate of 3 mL/min.

Incubations of VAS with RLMs

The RLMs were carefully thawed on ice before the experiment. VAS (200 μM) was mixed with alamethicin (25 μg/mg protein) and the microsomes (1.0 mg protein/mL) in 100 mM Tris-HCl

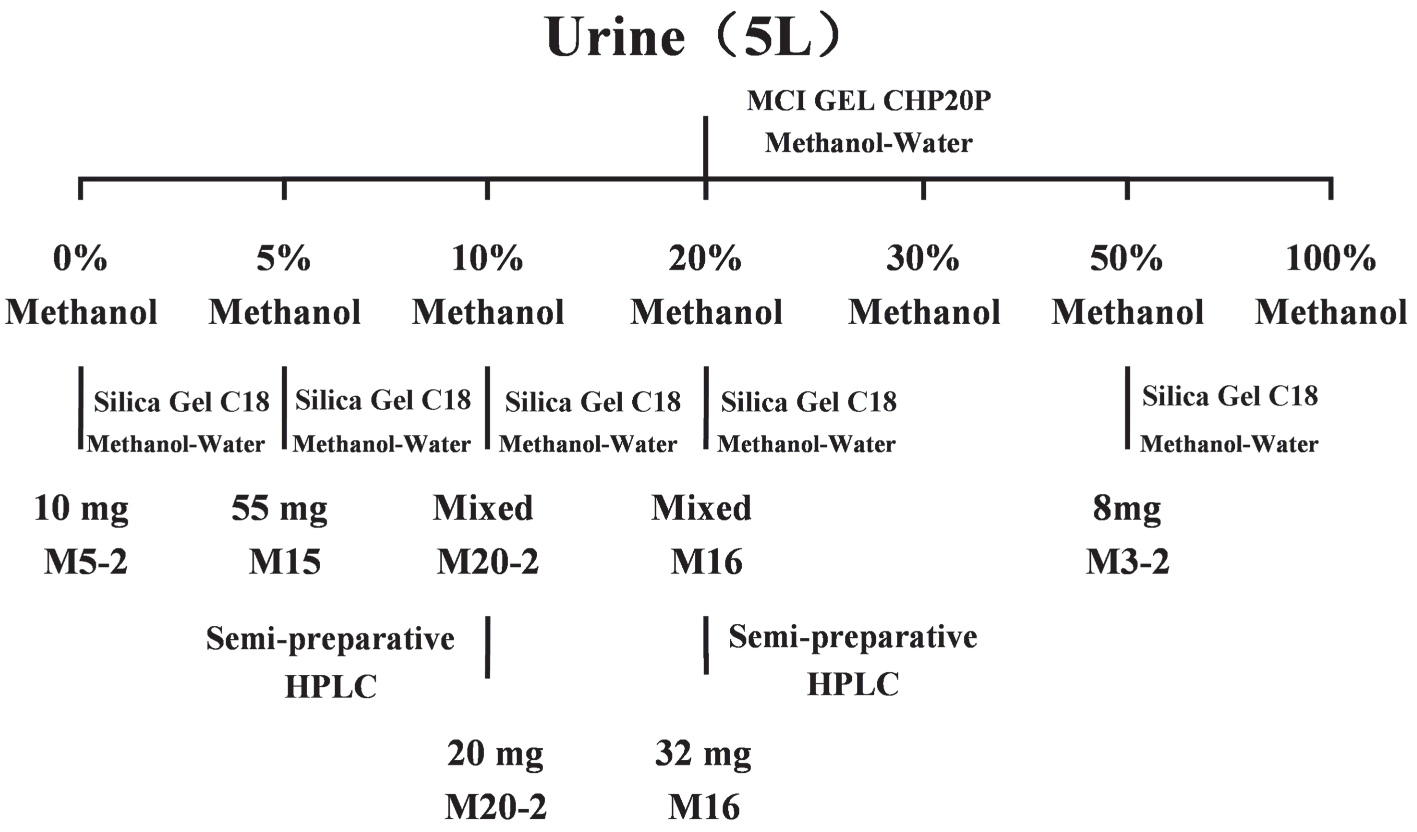

Fig 1. Metabolite isolation scheme of VAS from rat urine.

doi:10.1371/journal.pone.0122366.g001

buffer (50 mM, pH 7.4). After 5 min of pre-incubation at 37°C, the incubation reaction was initiated by the addition of glucose 6-phosphate (10 mM), glucose-6-phosphate dehydrogenase (1 unit/mL), NADPH (1.0 mM), and UDPGA (5.0 mM). The total incubation volume was 100 μL. The reactions were terminated with 300 μL ice-cold acetonitrile after 1 h of incubation. The mixture was centrifuged at 15,000 × *g* for 10 min, and then 320 μL of supernatant was evaporated to dryness by a gentle stream of nitrogen at 37°C. The residue was dissolved by 80 μL of 5% methanol and centrifuged at 15,000 × *g* for 10 min. The supernatant (5 μL) was then injected for UPLC/ESI-QTOF-MS system. Control samples without NADPH and UDPGA or substrates were prepared. The incubation was performed in duplicate.

Isolation, culture, and incubation of RPHs

Male rats (150 ± 20 g) were anesthetized with pentobarbital (100 mg/kg, ip) and their livers were perfused using the two-step collagenase digestion method described previously [27]. Cell vitality was assessed using the trypan blue method; only hepatocytes with a viability greater than 85% were seeded in a 6-well plate in M199 medium (Gibco, Laboratories Inc.; Grand Island, NY) supplemented with 5% fetal bovine serum, 10^{-9} mol/L dexamethasone, and insulin. Cells were cultured for 24 h before use in metabolism studies, and then added with VAS (200 μM) thereafter. After 1, 3, 5, 12, and 24 h of incubation, 100 μL supernatant was terminated with 300 μL of ice-cold acetonitrile. The mixture was centrifuged at 15,000 × *g* for 10 min, and 320 μL of supernatant was evaporated until dry by a gentle stream of nitrogen at 37°C. The

residue was dissolved by 80 μL of 5% methanol and centrifuged at 15,000 × *g* for 10 min. Up to 5 μL of the supernatant was injected into the UPLC/ESI-QTOF-MS system.

NMR instrumentation for identification of the metabolite structures

NMR spectra (^{1}H and ^{13}C NMR, HSQC, HMBC) were recorded on a Bruker AV 400 MHz spectrometer (400 MHz for ^{1}H and 100 MHz for ^{13}C). Chemical shifts were recorded in parts per million using tetramethylsilane as an internal standard. Deuterium reagents (DMSO-d6, CD_3OD, D_2O) were used as solvents for the metabolites.

In vitro anticholinesterase assays

The AChE and BChE inhibitory activities of VAS and its main metabolites, namely, VAO, VASL, VAOL, VAS-3-S, VAO-3-S, and VAS-3-G, were evaluated based on our previously established method [8]. A 50 μL incubation system composed of 10 μL of the test compound solution (all the test compound solutions were diluted to a series of concentrations with buffer solution before each experiment) and 40 μL of the enzyme solutions (with final concentrations: 0.0035 unit/mL for AChE, or 0.008 unit/mL for BChE) were mixed and pre-incubated for 15 min. Up to 50 μL of substrate solution (final concentration of 5.505 μM for ACh, or 7.152 μM for BCh) was added into the mixture, which was then incubated for 20 min at 25°C. The reaction was terminated by addition of 300 μL of ice acetonitrile solution (0°C), which was immediately dissolved with 1.899 μM IS (chlormequat). The solution was then centrifuged (15,000 × *g*, 10 min), and the supernatant was used for analysis by UPLC-ESI MS/MS. The inhibitive IC_{50} values of VAS and its key metabolites on AChE and BChE were calculated using the Prism software (GraphPad Software Inc., San Diego, CA).

UPLC Chromatography and Quadrupole Time-of-flight (Q/TOF) Mass Spectrometer

Chromatographic analysis was performed on an HSS T3 column (100 mm × 2.1 mm, 1.8 μm particle size; Waters Corporation, Milford, MA, USA) for separation of samples using an ACQUITY UPLC system equipped with a binary solvent manager system and an autosampler. The column was eluted with a gradient mobile phase of methanol (A) and 0.1% formic acid in deionized water (B): 0–6 min 5% A; 6–12 min, linear from 5% to 15% A; 12–18 min, linear from 15% to 30% A; 18–24 min, linear from 30% to 50% A; 24–28 min, linear from 50% to 90% A; 28–30 min, 5% A for equilibration of the column. The flow rate was 0.4 mL/min. The column and sample-tray temperatures were maintained at 40°C and 10°C, respectively.

A Waters ACQUITY Synapt G2 Q/TOF tandem mass spectrometer was combined with the UPLC system through an electrospray ionization (ESI) interface and controlled by Masslynx software (version 4.1, Waters Corp., Manchester, UK). The positive ionization mode of ESI source was operated. The optimized conditions for maximum detection of metabolites were as follows: source temperature, 120°C; desolvation temperature, 400°C; capillary voltage, 3.0 kV; sample cone, 30 V and extraction cone, 4.0 V. The cone and desolvation gas (N_2) flows were set at 75 and 800 (L/h), respectively. The leucine–enkephalin (2 ng/mL) was used as the lock mass to generate a reference ion in positive mode at *m/z* 556.2771 and the injection rate was 5 μL/min for accurate mass acquisition. Data were collected in centroid mode and the MS^E approach using a dynamic ramp of collision energy was carried out in two scan functions. Function 1 (low energy): mass scan ranged from 100–1000,with 0.2 s scan time and 0.05 s inter-scan delay; Function 2 (high energy): mass scan ranged from 100–1000 with 0.2 s scan time, 0.05 s inter-scan delay, and collision energy ramp of 10–40 V. MS/MS analysis were operated for major metabolites to obtain additional information from product ions since the same collision

energy of MS^E in Function 2 could not match each precursor ion. The conditions for the MS/MS functions, sample cone voltages, and sample collision energies were summarized in Table 1.

Results

Fragmentation of the parent VAS and important inter-metabolites (VAO, VASL, and VAOL)

Proper identification of metabolites using the on-line UPLC/QTOF-MS approach required comprehensive understanding of the fragmentation behavior of the parent compound and related compounds to be tested. Table 2 showed the elemental composition, experimental and calculated masses, and mass errors of the adduct ion and the fragment ions of VAS, VAO, VASL, and VAOL. The maximum mass errors between the measured and calculated values were less than 10 ppm ($\leq$ 2.0 mDa), which indicated high resolution and good accuracy.

Table 1. The optimized cone voltages (CV) and collision energies (CE) for protonated ions of vasicine and its main metabolites.

Metabolites	Precursor ions (m/z)	CV (V)	CE (V)
M0	189.1028	30	10–30
M1	203.0821	25	10–30
M2	203.1184	30	20–30
M3	205.0977	25	10–30
M4	217.0613	30	10–20
M5	219.0770	30	10–30
M6	219.1134	25	10–20
M7	221.0926	30	10–20
M8	235.0719	35	10–20
M9	235.1083	25	10–20
M10	237.0875	30	20–30
M11	245.1290	30	10–30
M12	247.1083	25	20–30
M13	251.1032	30	10–30
M14	261.1239	30	10–20
M15	269.0596	25	20–30
M16	283.0389	25	20–30
M17	285.0545	30	10–20
M18	297.0181	30	10–20
M19	299.0338	35	20–30
M20	365.1349	30	10–20
M21	379.1141	25	20–30
M22	381.1298	20	10–30
M23	393.0934	30	20–30
M24	395.1091	25	10–20
M25	397.1247	30	10–30
M26	411.1404	35	10–20
M27	413.1196	25	10–20
M28	421.1611	25	10–20
M29	437.1560	25	10–20
M30	557.1619	30	10–30

doi:10.1371/journal.pone.0122366.t001

Table 2. The predicted elemental compositions, measured masses and calculated masses, and mass errors of protonated vasicine, vasicinone, vasicinol, vasicinolone and their fragment ions.

Compounds	Fragment ions elemental composition	Measured mass (Da)	Calculated mass (Da)	Error (mDa)	Error (ppm)
VAS	$C_{11}H_{13}N_2O^+$	189.1030	189.1028	0.2	1.1
	$C_{11}H_{11}N_2^+$	171.0925	171.0922	0.3	1.8
	$C_{11}H_8N^+$	154.0657	154.0657	0.0	0.0
	$C_{10}H_{10}N^+$	144.0812	143.0813	-0.1	-0.7
	$C_{10}H_9N^+$	143.0736	143.0735	0.1	0.7
	$C_8H_8N^+$	118.0663	118.0657	0.6	5.1
	$C_7H_7^+$	91.0555	91.0548	0.7	7.7
VAO	$C_{11}H_{11}N_2O_2^+$	203.0824	203.0821	0.3	1.5
	$C_{11}H_9N_2O^+$	185.0716	185.0715	0.1	0.5
	$C_{11}H_7N_2^+$	167.0620	167.0609	1.1	6.6
	$C_{10}H_9N_2^+$	157.0758	157.0766	-0.8	-5.1
	$C_{10}H_6N^+$	140.0502	140.0500	0.2	1.4
	$C_9H_8N^+$	130.0646	130.0657	-1.1	-8.5
VASL	$C_{11}H_{13}N_2O_2^+$	205.0971	205.0977	-0.6	-2.9
	$C_{11}H_{11}N_2O^+$	187.0882	187.0871	1.1	5.9
	$C_{11}H_9N_2O^+$	185.0730	185.0715	0.7	3.8
	$C_{11}H_9N_2^+$	169.0775	169.0766	0.9	5.3
	$C_{10}H_{10}NO^+$	160.0755	160.0762	-0.7	-4.4
	$C_{10}H_9NO^+$	159.0683	159.0684	-0.6	-3.8
	$C_8H_8NO^+$	134.0618	134.0606	0.9	6.7
VAOL	$C_{11}H_{11}N_2O_3^+$	219.0781	219.0770	1.1	5.0
	$C_{11}H_9N_2O_2^+$	201.0672	201.0664	0.8	4.0
	$C_{11}H_7N_2O^+$	183.0568	183.0558	1.0	5.5
	$C_{10}H_9N_2O^+$	173.0726	173.0715	1.1	6.4
	$C_{10}H_6NO^+$	156.0454	156.0449	0.5	3.2
	$C_9H_8NO^+$	146.0610	146.0606	0.4	2.7

doi:10.1371/journal.pone.0122366.t002

The dynamic energy of MS^2 from protonated VAS $[M+H]^+$ at *m/z* 189.1030 afforded the product ion at *m/z* 171.0925 by the loss of water molecules (18.0105 Da, exacted 18.0106 Da). As a result, a carbon–carbon double bond between C-2 and C-3 through elimination reaction was formed (Fig 2A). Thereafter, the product ion at *m/z* 154.0657 ($C_{11}H_8N^+$, calculated *m/z* 154.0657), 144.0812 ($C_{10}H_{10}N^+$, calculated *m/z* 144.0813), and 143.0726 ($C_{10}H_9N^+$, calculated *m/z* 143.0735) were formed through the breakdown of rings B and C, as well as the elimination of ammonia (NH_3, 17.0268 Da, exact 17.0265 Da), CHN unit (27.0113 Da, exact 27.0109 Da), and CH_2N radicals (28.0189 Da, exact 28.0187 Da). The fragmentation process was similar to that reported by Rashkes, who studied the fragmentation of natural and synthetic quinazoline derivatives [28]. In addition, a heptatomic and octatomic ring was produced in the fragmentation of natural and synthetic quinazoline derivatives (Fig 2A). The subsequent successive neutral loss of molecule in the C_2H_2 unit (26.0149 Da, exact 26.0155) (from C-4) and in the C_2H_2 and CHN units (53.0257 Da, exact 52.0265 Da) from the ion at *m/z* 144 generated the product ion at *m/z* 118.0663 ($C_8H_8N^+$, calculated *m/z* 118.0657) and 91.0555 ($C_7H_7^+$, calculated *m/z* 91.0548). The fragmentation pathways of VAS were showed in Fig 2A.

VAO is a metabolite of VAS [2] resulting from the ketonization of VAS in C-9 site. However, the fragmentation pathways of VAO were different from VAS. ESI-MS spectral analysis of VAO revealed a protonated molecule ion at *m/z* 203.0824. The MS^2 spectra showed identical

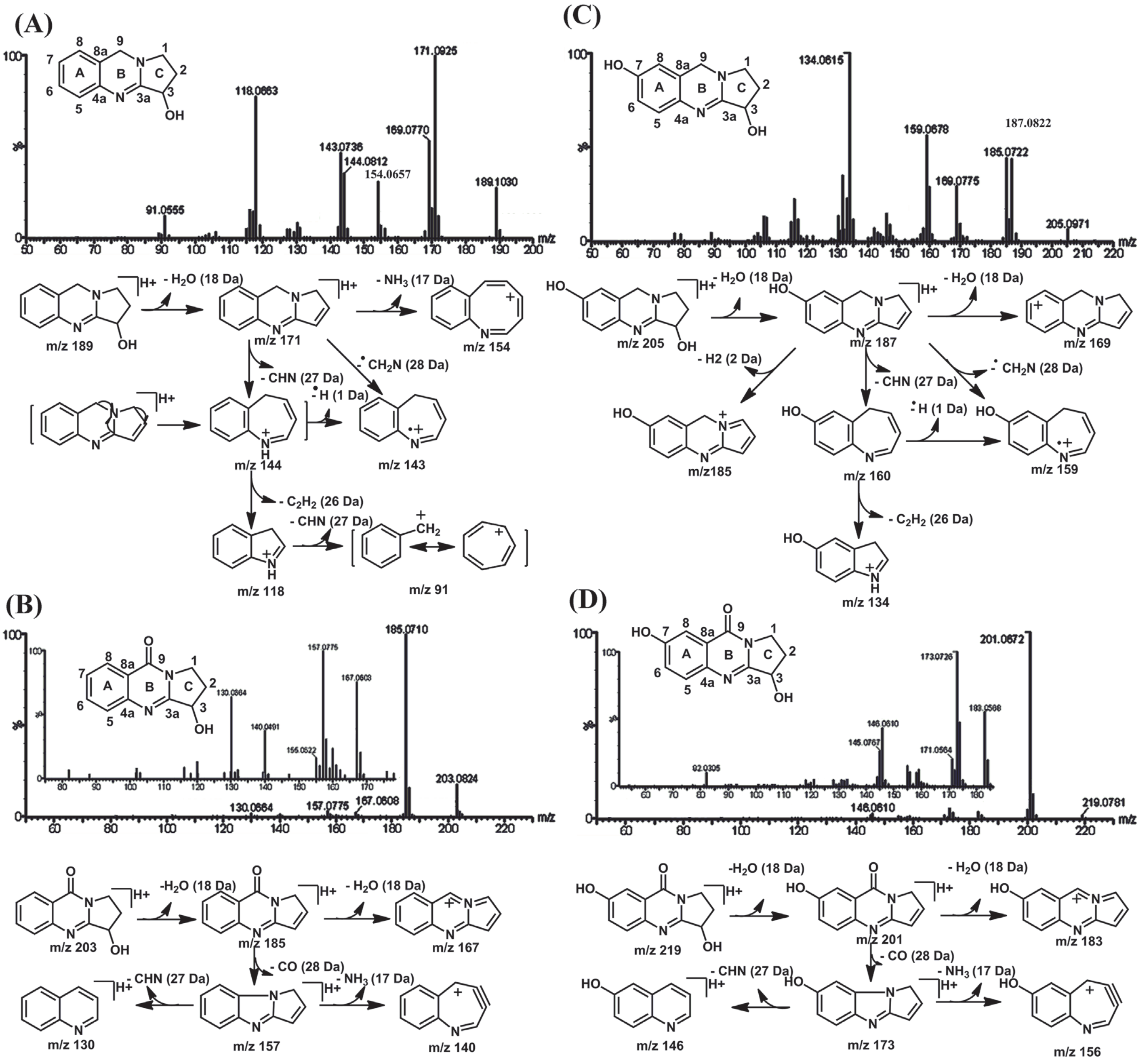

Fig 2. UPLC/ESI-QTOF-MS spectra of VAS (A), vasicinol (B), VAO (C), and vasicinolone (D) using ESI in positive ion mode.

doi:10.1371/journal.pone.0122366.g002

fragment ions at *m/z* 185.0726 ($C_{11}H_9N_2O^+$, calculated *m/z* 185.0715), 167.0620 ($C_{11}H_7N_2^+$, calculated *m/z* 167.0616), 157.0758 ($C_{10}H_9N_2^+$, calculated *m/z* 157.0766), 140.0502 ($C_{10}H_6N^+$, calculated *m/z* 140.0500), and 130.0546 ($C_9H_8N^+$, calculated *m/z* 130.0557). The fragment ions were acquired from successive loss of a H_2O (18 Da); molecules of two H_2O (36 Da); molecules of H_2O and a neutral loss of CO (46 Da); molecules of H_2O, a neutral loss of CO and NH_3 (63 Da); loss of molecules of H_2O, a neutral loss of CO, and CHN unit (73 Da). The fragmentation pathways of VAO were showed in Fig 2B.

VASL is a hydroxylation metabolite of VAS at C-7. VASL exhibited a $[M+H]^+$ ion at *m/z* 205.0971, which was 16 Da heavier than VAS. The MS^2 spectra showed identical fragment ions

at *m/z* 187.0882 ($C_{11}H_{11}N_2O^+$, calculated *m/z* 187.0871), 185.0730 ($C_{11}H_9N_2O^+$, calculated *m/z* 185.0715), 169.0775 ($C_{11}H_9N_2^+$, calculated *m/z* 169.0766), 160.0755 ($C_{10}H_{10}NO^+$, calculated *m/z* 160.0762), 159.0683 ($C_{10}H_9NO^+$, calculated *m/z* 159.0684), and 134.0618 ($C_8H_8NO^+$, calculated *m/z* 134.0606). The fragment ions were acquired from the successive loss of H_2O (18 Da), molecules of H_2O and 2H (20 Da), molecules of two H_2O (36 Da), molecules of H_2O and a neutral loss of the CHN unit (45 Da), molecules of H_2O, and CH_2N radicals (46 Da), loss of H_2O molecules, and a neutral loss of CHN and C_2H_2 (71 Da). The fragmentation pathways of VASL were showed in Fig 2C. The fragmentation pathways of VASL were very similar with that of VAS, which was accustomed to the successive loss of H_2O, CHN unit, CH_2N radicals, and the C_2H_2 unit. Furthermore, the special fragment ion at *m/z* 134.0618 ($C_8H_8NO^+$, calculated *m/z* 134.0606) showed a hydroxyl group of ring A, which could be used to identify the metabolite that undergoes hydroxylation in ring A.

VAOL is a hydroxylation metabolite of VAO at C-7. VAOL exhibited a $[M+H]^+$ ion at *m/z* 219.0781, which was 16 Da heavier than VAO. The MS^2 spectra showed identical fragment ions at *m/z* 201.0672 ($C_{11}H_9N_2O_2^+$, calculated *m/z* 201.0664), 183.0568 ($C_{11}H_7N_2O^+$, calculated *m/z* 183.0558), 173.0726 ($C_{10}H_9N_2O^+$, calculated *m/z* 173.0715), 156.0454 ($C_{10}H_6NO^+$, calculated *m/z* 156.0449), and 146.0610 ($C_9H_8NO^+$, calculated *m/z* 146.0606). The fragment ions were acquired from the successive loss of H_2O (18 Da), molecules of two H_2O (36 Da), molecules of H_2O and a neutral loss of CO (46 Da), molecule of H_2O, a neutral loss of CO, NH_3 (63 Da), loss of H_2O molecule, and a neutral loss of CO and the CHN unit (73 Da). The fragmentation pathways of VAOL were presented in Fig 2D. The fragmentation pathways of VAOL were very similar to that of VAO, which is accustomed to the successive loss of H_2O, CO unit and the CHN unit and NH_3. The CO unit could be used to identify the metabolites, whose parent compound undergoes ketonization. Furthermore, the special fragment ion at 146.0610 ($C_9H_8NO^+$, calculated *m/z* 146.0606) showed a hydroxyl group of ring A, which could be used to identify the metabolites that undergoes hydroxylation in ring A.

Characterization of the metabolites in vivo and in vitro

Figs 3–8 showed the extracted and total ion chromatograms of the parent compound and their metabolites in vivo (rat urine, feces, bile, plasma) and in vitro (RLMs and RPHs). A total of 72 metabolites were observed in rat urine, and 15, 45, 25, 18, and 11 metabolites were found in vivo in rat feces, bile, and plasma and in vitro in RLMs and RPHs, respectively. The metabolites were numbered according to their molecular weight and chromatographic retention times. Table 3 listed the detailed information (including of retention times, presume formula, measured mass, calculated mass, fragment ions and presence in different samples) of the metabolites. The structures of metabolite were presumed by the comparison of mass spectra and chromatographic retention times with available reference standards, or by its mass spectral fragmentation patterns. The proposed structures of the metabolites were showed in Fig 9, which included monohydroxylation, dihydroxylation, trihydroxylation, dehydrogenation, methylation, acetylation, sulfation, and glucuronation of the parent compound.

Metabolite M1. Metabolite M1 (retention time t_R = 13.90 min) is a metabolite detected in the in vivo and in vitro samples. Metabolite M1 exhibited a $[M + H]^+$ ion at *m/z* 203.0839, which was 14 Da (13.9807 Da) higher than that of the protonated parent compound, indicating the introduction of an oxygen atom and dehydrogenation (+ 13.9793 Da). The metabolic site for the parent compound may be at C-1, C-2, or C-9. The MS^2 spectra showed identical fragment ions at *m/z* 185.0725 ($C_{11}H_9N_2O^+$, calculated *m/z* 185.0715), 167.0603 ($C_{11}H_7N_2^+$, calculated *m/z* 167.0609), 157.0775 ($C_{10}H_9N_2^+$, calculated *m/z* 157.0766), 140.0509 ($C_{10}H_6N^+$, calculated *m/z* 140.0500), and 130.0654 ($C_9H_8N^+$, calculated *m/z* 130.0657). Our data indicated

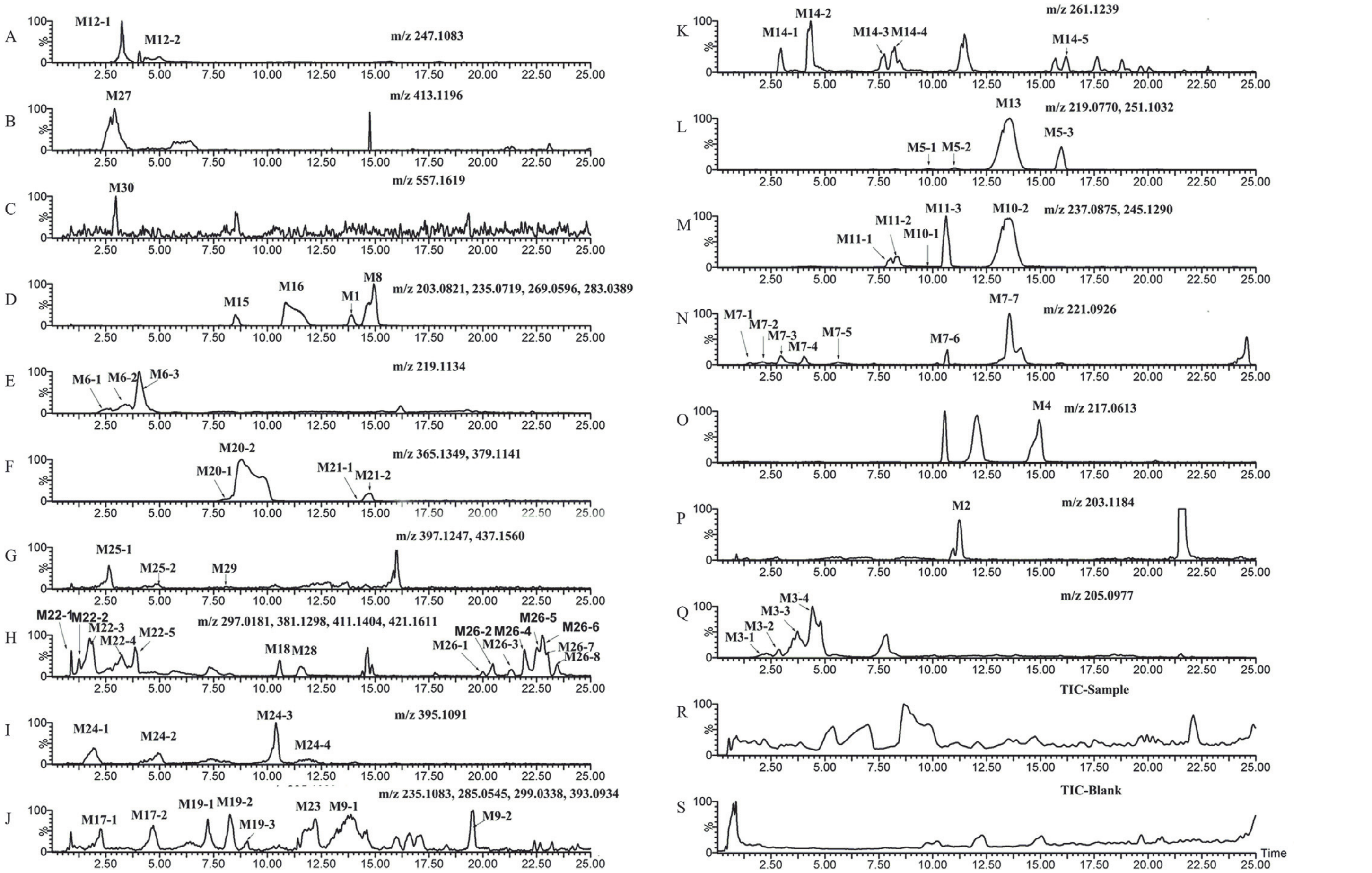

Fig 3. The total ion chromatogram (TIC) and extract ion chromatograms (EIC) of urine in vivo after oral administration of 20 mg/kg VAS. A–Q: EIC of sample urine. R: TIC of sample urine. S: TIC of blank urine.

doi:10.1371/journal.pone.0122366.g003

that M1 may undergo successive loss of H_2O (18 Da), molecules of two H_2O (36 Da), molecules of a H_2O and a neutral loss of CO (46 Da), molecules of a H_2O, a neutral loss of CO and NH_3 (63 Da), loss of molecules of a H_2O, and a neutral loss of CO and the CHN unit (73 Da). Our conclusion was confirmed by comparing the retention time and MS^2 spectra with those of the authentic standard (VAO).

Metabolite M2. Metabolite M2 (retention time t_R = 11.28 min) exhibited a $[M + H]^+$ ion at *m/z* 203.1190, which was 14 Da (14.0162 Da) higher than that of the protonated parent compound, indicating the introduction of methyl group (+ 14.0156 Da). Metabolite M2 was only acquired in urine. The MS^2 spectra showed identical fragment ions at *m/z* 187.0886 ($C_{11}H_{11}N_2O^+$, calculated *m/z* 187.0871), 169.0783 ($C_{11}H_9N_2^+$, calculated *m/z* 169.0766), 160.0650 ($C_9H_8N_2O^+$, calculated *m/z* 160.0637), 159.0567 ($C_9H_7N_2O^+$, calculated *m/z* 159.0558), and 131.0625 ($C_8H_7N_2^+$, calculated *m/z* 131.0609). Given the clearance of the methyl group during the first step, no further information could help determine the position of the methyl group; thus, the position of the methyl group was unclear.

Metabolites M3. M3 was detected with a protonated molecular weight of 205.0977, which was 16 Da (15.9949 Da) higher than that of the protonated parent compound, indicating the introduction of one hydroxylation metabolite. In addition, four independent chromatographic peaks with protonated ions at *m/z* 205.0977 were detected.

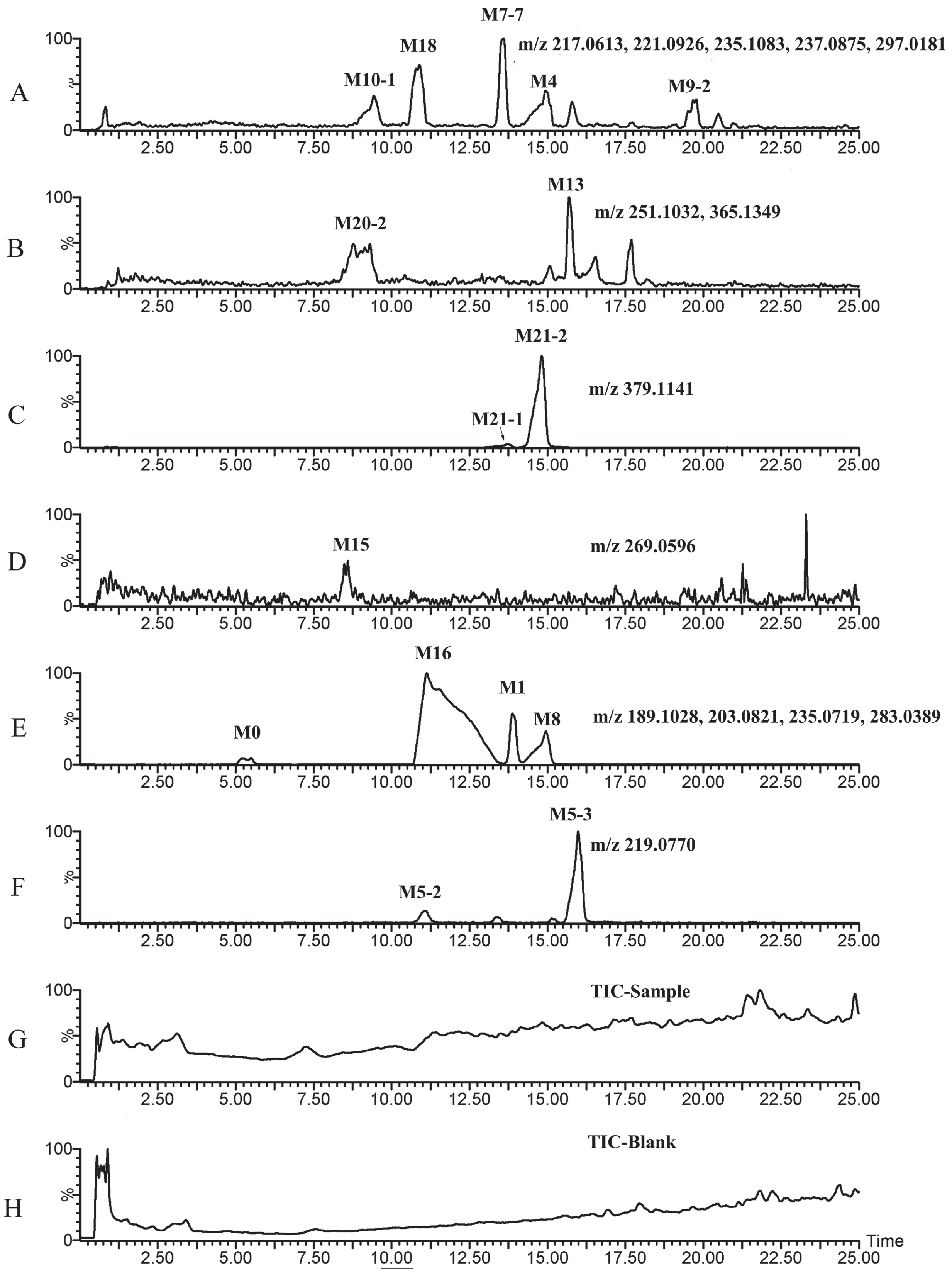

Fig 4. TIC and EIC of feces in vivo after oral administration of 20 mg/kg VAS. A–F: EIC of sample feces. G: TIC of sample feces. H: TIC of blank feces.

doi:10.1371/journal.pone.0122366.g004

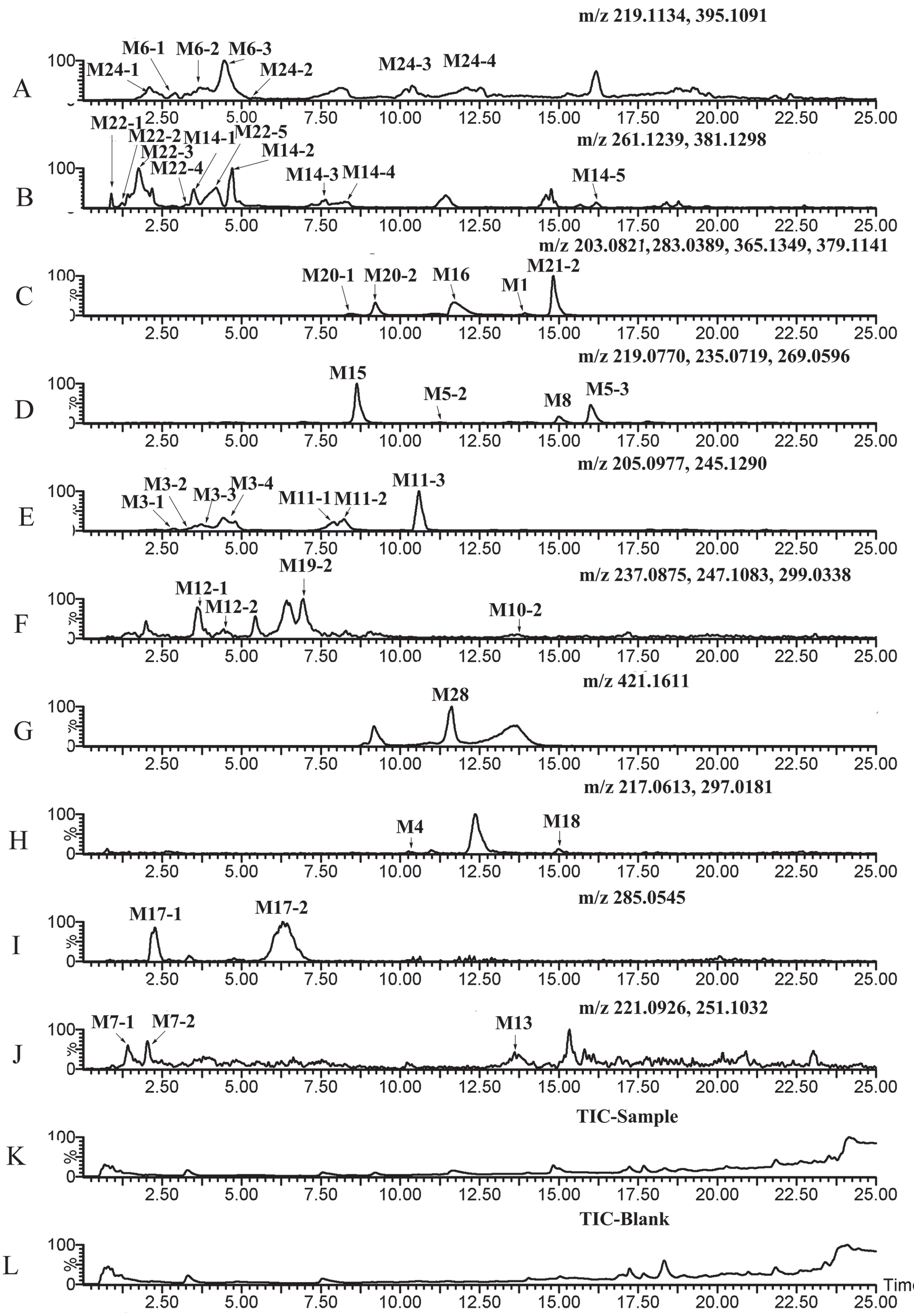

Fig 5. TIC and EIC of bile in vivo after oral administration of 20 mg/kg VAS. A–J: EIC of sample bile. K: TIC of sample bile. L: TIC of blank bile.

doi:10.1371/journal.pone.0122366.g005

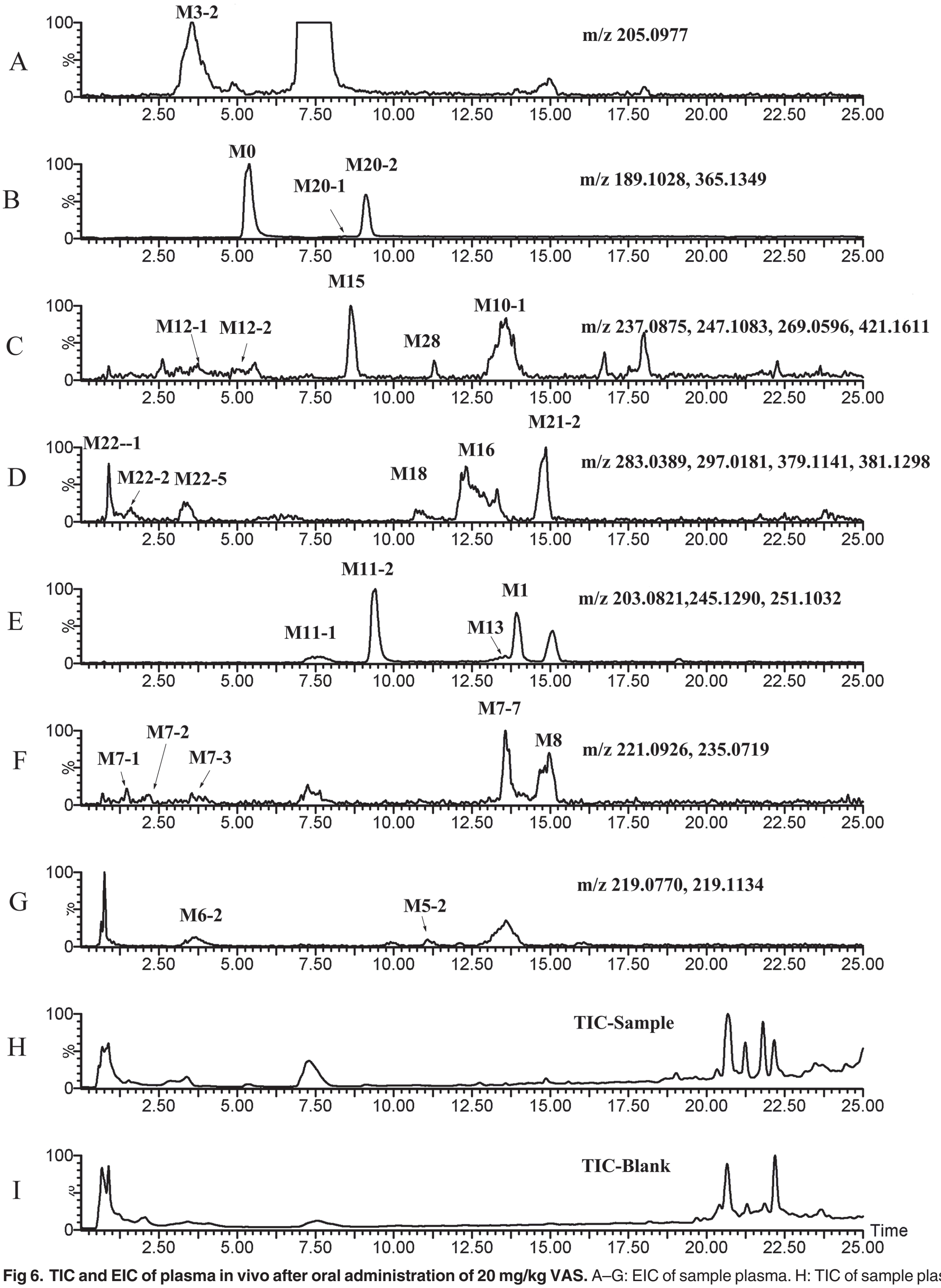

Fig 6. TIC and EIC of plasma in vivo after oral administration of 20 mg/kg VAS. A–G: EIC of sample plasma. H: TIC of sample plasma. I: TIC of blank plasma.

doi:10.1371/journal.pone.0122366.g006

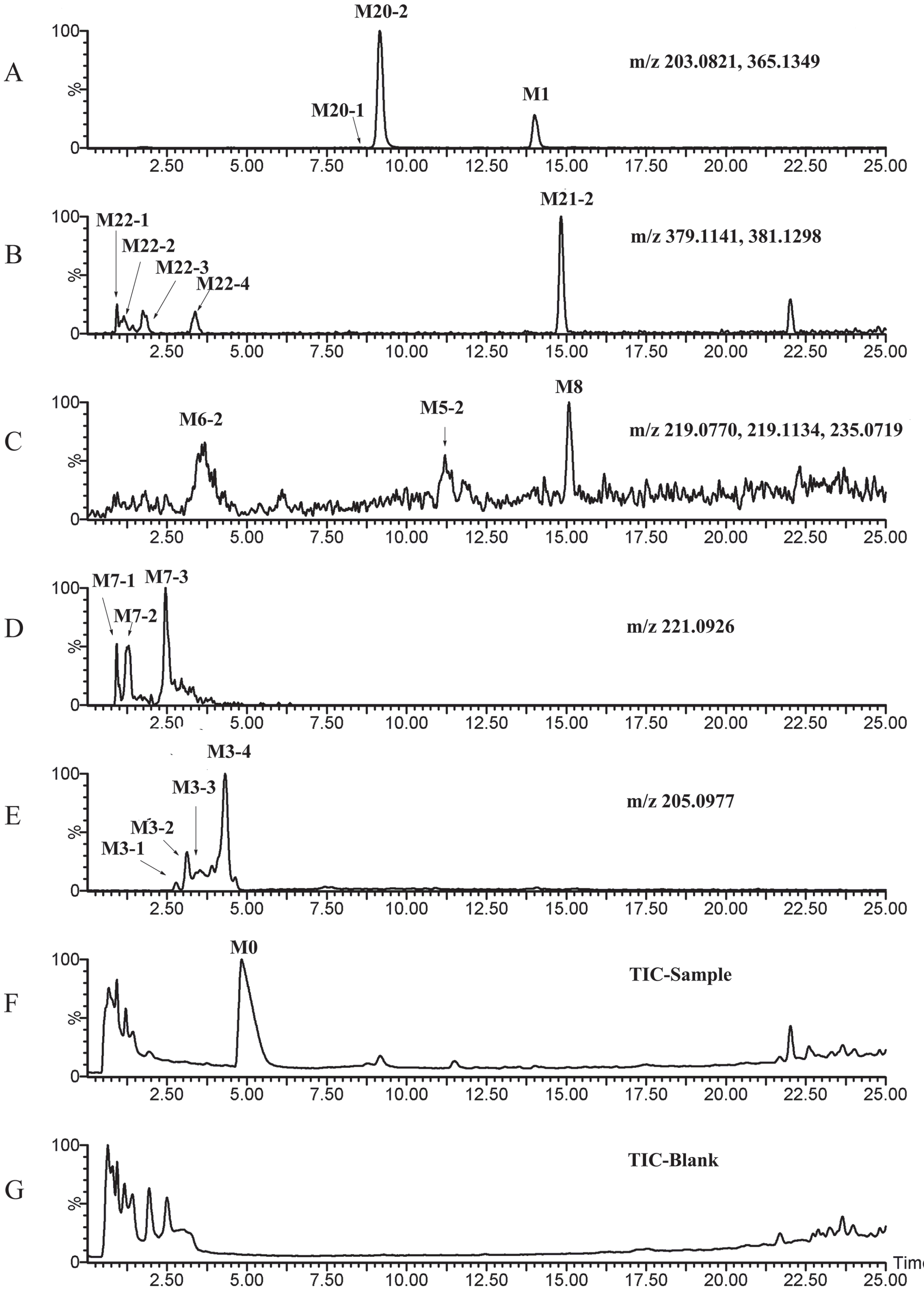

Fig 7. TIC and EIC of RLMs in vitro after incubation of 200 μM VAS. A–E: EIC of sample RLMs. F: TIC of sample RLMs. G: TIC of blank RLMs.

doi:10.1371/journal.pone.0122366.g007

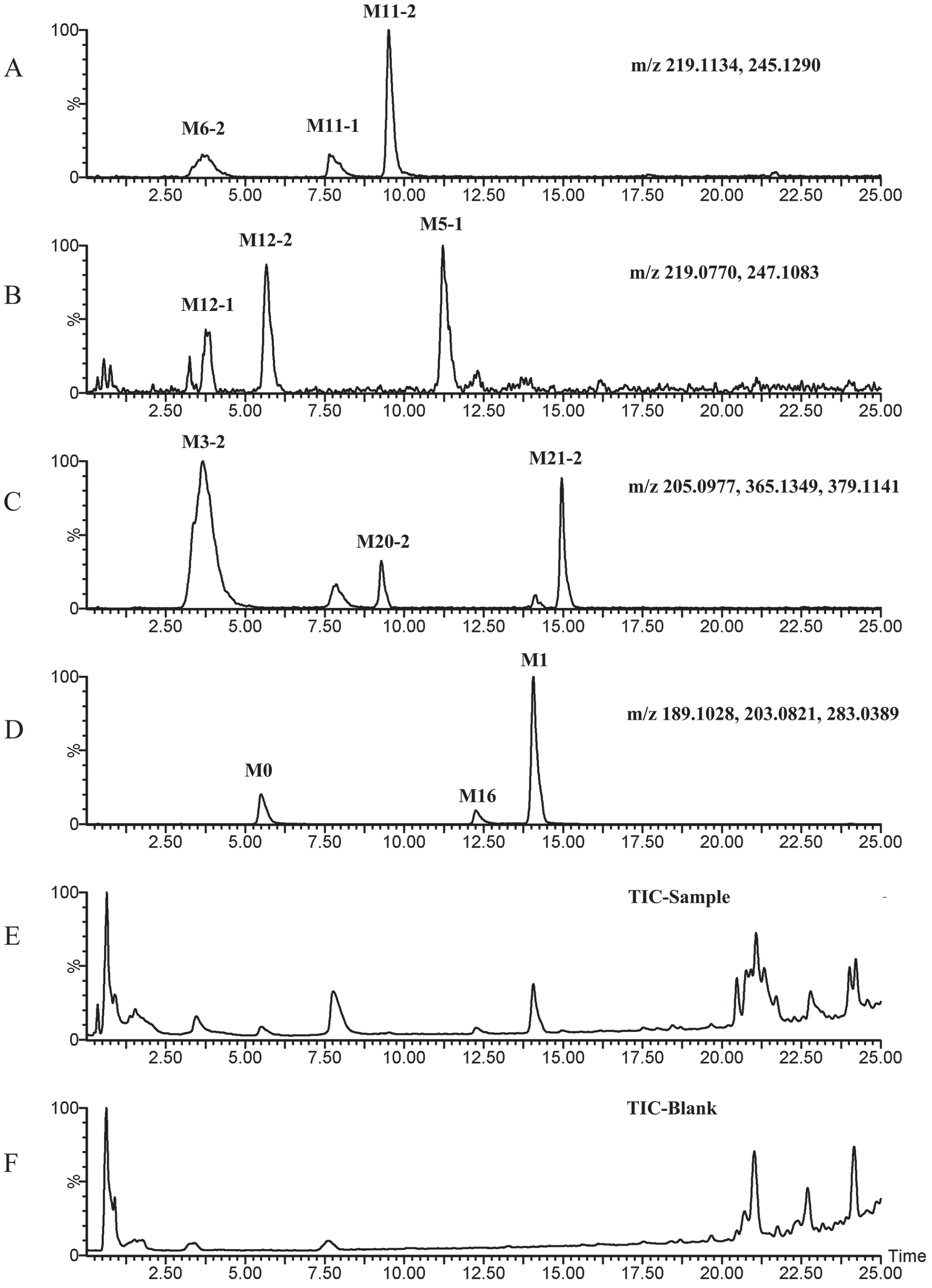

Fig 8. TIC and EIC of RPHs in vitro after incubation of 200 μM VAS. A–D: EIC of sample RPHs. E: TIC of sample RPHs. F: TIC of blank RPHs.

doi:10.1371/journal.pone.0122366.g008

Table 3. Characterization of metabolites of vasicine *in vivo* and *in vitro* in rat by UPLC/Q-TOF.

Metabolits	Description	RT (min)	Formula	Measured mass	Calculated mass	Fragment ions	U	F	B	P	L	H
M0	parent	5.37	$C_{11}H_{13}N_2O$	189.1030	189.1028	171.0925, 154.0657, 144.0812, 143.0726, 118.0663, 91.0555	+	+	-	+	+	+
M1	hydroxyl +dehydrogen	13.90	$C_{11}H_{11}N_2O_2$	203.0839	203.0821	185.0725, 167.0603, 157.0775, 140.0509, 130.0654	+	+	+	+	+	+
M2	methyl	11.28	$C_{12}H_{15}N_2O$	203.1190	203.1184	187.0884, 169.0774, 160.0643, 159.0567, 131.0619	+	-	-	-	-	-
M3-1	monohydroxyl	2.58	$C_{11}H_{13}N_2O_2$	205.0962	205.0977	187.0875, 169.0751, 159.0922, 130.0651	+	+	+	-	+	-
M3-2	monohydroxyl	2.81	$C_{11}H_{13}N_2O_2$	205.0970	205.0977	187.0875, 185.0722, 169.0789, 160.0761, 159.0679, 134.0606	+	+	+	-	+	-
M3-3	monohydroxyl	3.53	$C_{11}H_{13}N_2O_2$	205.0978	205.0977	187.0865, 169.0780, 159.0853, 130.0662	+	+	+	+	+	+
M3-4	monohydroxyl	3.79	$C_{11}H_{13}N_2O_2$	205.0989	205.0977	187.0879, 169.0775, 161.0715, 133.0766,106.0675	+	+	+	-	+	-
M4	dihydroxyl +didehydrogen	10.56	$C_{11}H_9N_2O_3$	217.0606	217.0613	199.0500, 189.0654, 160.0390, 147.0561, 144.0445, 132.0453	+	+	+	-	-	-
M5-1	dihydroxyl +dehydrogen	10.01	$C_{11}H_{11}N_2O_3$	219.0771	219.0770	201.0652, 183.0562, 173.0703, 160.0674, 132.0448	+	+	-	+	+	+
M5-2	dihydroxyl +dehydrogen	11.27	$C_{11}H_{11}N_2O_3$	219.0779	219.0770	201.0675, 183.0569, 173.0724,146.0589	+	+	+	+	-	-
M5-3	hydroxyl +dehydrogen +N-oxyl	16.18	$C_{11}H_{11}N_2O_3$	219.0788	219.0770	201.0673, 173.0728, 155.0625, 130.0668	+	-	+	+	-	-
M6-1	methoxyl	2.10	$C_{12}H_{15}N_2O_2$	219.1149	219.1134	187.0825, 171.0934, 158.0621, 132.0438, 130.0676	+	-	+	-	-	-
M6-2	methoxyl	3.46	$C_{12}H_{15}N_2O_2$	219.1150	219.1134	187.0875, 171.0935, 160.0648, 159.0581, 131.0618	+	-	+	+	+	+
M6-3	methoxyl	4.04	$C_{12}H_{15}N_2O_2$	219.1153	219.1134	187.0892, 171.0936, 160.0659, 159.0583, 131.0631	+	-	+	-	-	-
M7-1	dihydroxyl	1.48	$C_{11}H_{13}N_2O_3$	221.0932	221.0926	203.0825, 175.0896, 158.0601, 157.0771, 130.0669	+	-	+	-	+	-
M7-2	dihydroxyl	2.62	$C_{11}H_{13}N_2O_3$	221.0936	221.0926	203.0327, 185.0724, 161.0727, 133.0779	+	-	+	-	+	-
M7-3	dihydroxyl	3.60	$C_{11}H_{13}N_2O_3$	221.0925	221.0926	203.0820, 185.0700, 161.0710, 133.0767	+	-	-	-	+	-
M7-4	dihydroxyl	4.12	$C_{11}H_{13}N_2O_3$	221.1069	221.0926	203.0857, 185.0732, 161.0715, 133.0763	+	-	-	-	-	-
M7-5	monohydroxyl +N-oxyl	5.81	$C_{11}H_{13}N_2O_3$	221.0921	221.0926	203.0848, 185.0754, 175.0836, 146.0845, 130.0696	+	-	-	-	-	-
M7-6	monohydroxyl +N-oxyl	10.80	$C_{11}H_{13}N_2O_3$	221.0940	221.0926	203.0859, 185.0735, 173.0730, 160.0419, 132.0435	+	-	-	-	-	-
M7-7	monohydroxyl +N-oxyl	13.59	$C_{11}H_{13}N_2O_3$	221.0917	221.0926	203.0830, 185.0702, 173.0709, 155.0608, 130.0674	+	-	-	+	-	-
M8	trihydroxyl +dehydrogen	14.94	$C_{11}H_{11}N_2O_4$	235.0727	235.0719	217.0623, 199.0516, 175.0722, 173.0519, 171.0567, 147.0567	+	+	+	+	+	-
M9-1	dihydroxyl +methyl	13.92	$C_{12}H_{15}N_2O_3$	235.1084	235.1083	NA	+	-	-	+	-	-
M9-2	dihydroxyl +methyl	19.56	$C_{12}H_{15}N_2O_3$	235.1067	235.1083	NA	+	-	-	-	-	-
M10-1	trihydroxyl	9.81	$C_{11}H_{13}N_2O_4$	237.0890	237.0875	219.0741, 201.0718, 189.0674, 160.0666, 146.0618	+	+	-	-	-	-
M10-2	trihydroxyl	13.36	$C_{11}H_{13}N_2O_4$	237.0876	237.0875	219.0774, 201.0670, 183.0563, 159.0571, 157.0770, 132.0459	+	+	+	+	-	-
M11-1	acetyl+methyl	8.05	$C_{14}H_{17}N_2O_2$	245.1290	245.1290	203.1181, 187.0885, 169.0785, 160.0647, 159.0602, 131.0621	+	+	+	+	-	+
M11-2	acetyl+methyl	8.38	$C_{14}H_{17}N_2O_2$	245.1287	245.1290	203.1185, 187.0891, 169.0775, 160.0655, 159.0609, 131.0623	+	+	+	+	-	-
M11-3	acetyl+methyl	10.63	$C_{14}H_{17}N_2O_2$	245.1291	245.1290	203.1197, 187.0887, 169.0781, 160.0649, 159.0601, 131.0624	+	+	+	+	-	+
M12-1	hydroxyl+acetyl	4.00	$C_{13}H_{15}N_2O_3$	247.1054	247.1083	230.0955, 187.0849, 169.0758, 159.0585, 131.0608	+	+	+	+	-	+
M12-2	hydroxyl+acetyl	4.42	$C_{13}H_{15}N_2O_3$	247.1059	247.1083	229.0963, 187.0848, 169.0753, 160.0619, 131.0607	+	+	+	+	-	+
M13	dihydroxyl +methoxyl	13.47	$C_{12}H_{15}N_2O_4$	251.1025	251.1032	219.0755, 201.0676, 183.0570,157.0786	+	+	+	+	-	-
M14-1	hydroxyl+acetyl +methyl	2.65	$C_{14}H_{17}N_2O_3$	261.1245	261.1239	244.1195, 188.0939, 187.0882, 160.0641, 144.0700, 131.0616	+	-	+	-	-	-

(*Continued*)

Table 3. (*Continued*)

Metabolits	Description	RT (min)	Formula	Measured mass	Calculated mass	Fragment ions	U	F	B	P	L	H
M14-2	hydroxyl+acetyl +methyl	4.48	$C_{14}H_{17}N_2O_3$	261.1245	261.1239	244.1176, 226.1070, 188.0937, 187.0865, 169.0765, 160.0647	+	+	+	-	-	-
M14-3	hydroxyl+acetyl +methyl	7.89	$C_{14}H_{17}N_2O_3$	261.1166	261.1239	231.1183, 188.0937, 160.0637, 144.0690 121.0668	+	-	+	-	-	-
M14-4	hydroxyl+acetyl +methyl	8.38	$C_{14}H_{17}N_2O_3$	261.1213	261.1239	244.1195, 188.0962, 187.0878, 160.0650, 144.0700, 131.0615	+	-	+	-	-	-
M14-5	hydroxyl+acetyl +methyl	15.76	$C_{14}H_{17}N_2O_3$	261.1221	261.1239	NA	+	-	+	-	-	-
M15	sulf	8.53	$C_{11}H_{13}N_2SO_4$	269.0609	269.0596	189.1038, 171.0932, 154.0672, 144.0824, 118.0668	+	+	+	+	-	-
M16	hydroxyl +dehydrogen +sulf	10.82	$C_{11}H_{11}N_2SO_5$	283.0413	283.0389	203.0826. 185.0725, 167.0624, 157.0767, 140.0529, 130.0653	+	+	+	+	-	+
M17-1	hydroxyl+sulf	2.24	$C_{11}H_{13}N_2SO_5$	285.0545	285.0545	205.0981, 187.0880, 160.0617, 134.0694	+	-	+	-	-	-
M17-2	hydroxyl+sulf	4.69	$C_{11}H_{13}N_2SO_5$	285.0518	285.0545	205.0988, 187.0890, 160.0673, 134.0626	+	+	+	-	-	-
M18	dihydroxyl +didehydrogen +sulf	10.56	$C_{11}H_9N_2SO_6$	297.0185	297.0181	217.0618, 199.0539, 160.0400. 147.0590	+	+	+	+	-	-
M19-1	dihydroxyl +dehydrogen +sulf	6.37	$C_{11}H_{11}N_2SO_6$	299.0341	299.0338	219.0748	+	-	-	-	-	-
M19-2	dihydroxyl +dehydrogen +sulf	7.22	$C_{11}H_{11}N_2SO_6$	299.0313	299.0338	219.0766	+	-	+	-	-	-
M19-3	dihydroxyl +dehydrogen +sulf	8.25	$C_{11}H_{11}N_2SO_6$	299.0313	299.0338	219.0766	+	+	-	-	-	-
M20-1	glucuronid	7.90	$C_{17}H_{21}N_2O_7$	365.1321	365.1349	189.1023, 171.0924, 154.0644, 144.0838, 118.0649	+	+	+	-	+	-
M20-2	glucuronid	8.81	$C_{17}H_{21}N_2O_7$	365.1345	365.1349	189.1033, 171.0928, 154.0650, 144.0824, 118.0670	+	+	+	+	+	+
M21-1	hydroxyl +dehydrogen +glucuronid	13.43	$C_{17}H_{19}N_2O_8$	379.1154	379.1141	203.0836, 185.0724	+	+	-	-	-	-
M21-2	hydroxyl +dehydrogen +glucuronid	14.74	$C_{17}H_{19}N_2O_8$	379.1143	379.1141	203.0829, 185.0722	+	+	+	+	+	+
M22-1	hydroxyl +glucuronid	0.89	$C_{17}H_{21}N_2O_8$	381.1266	381.1298	205.0983, 187.0876, 160.1437, 134.0598	+	-	+	-	+	-
M22-2	hydroxyl +glucuronid	1.25	$C_{17}H_{21}N_2O_8$	381.1310	381.1298	205.0976, 187.0874, 166.0770, 134.0643	+	-	+	-	+	-
M22-3	hydroxyl +glucuronid	1.75	$C_{17}H_{21}N_2O_8$	381.1281	381.1298	205.0977, 187.0842,166.0818, 134.0596	+	-	+	-	+	-
M22-4	hydroxyl +glucuronid	3.24	$C_{17}H_{21}N_2O_8$	381.1290	381.1298	205.0979, 187.0871, 160.0415, 134.0580	+	-	+	+	+	-
M22-5	hydroxyl +glucuronid	3.84	$C_{17}H_{21}N_2O_8$	381.1305	381.1298	205.0976, 187.0876, 160.0425, 136.0780	+	-	+	-	-	-
M23	dihydroxyl +didehydrogen +glucuronid	12.25	$C_{17}H_{17}N_2O_9$	393.0943	393.0934	217.0611, 199.0509, 160.0600, 147.0548	+	-	-	-	-	-
M24-1	dihydroxyl +dehydrogen +glucuronid	1.91	$C_{17}H_{19}N_2O_9$	395.1102	395.1091	219.0778, 201.0659	+	-	+	-	-	-

(*Continued*)

Table 3. (*Continued*)

Metabolits	Description	RT (min)	Formula	Measured mass	Calculated mass	Fragment ions	U	F	B	P	L	H
M24-2	dihydroxyl +dehydrogen +glucuronid	4.92	$C_{17}H_{19}N_2O_9$	395.1120	395.1091	219.0788, 201.0670	+	-	+	-	-	-
M24-3	dihydroxyl +dehydrogen +glucuronid	10.40	$C_{17}H_{19}N_2O_9$	395.1074	395.1091	219.0775, 201.0666	+	-	+	-	-	-
M24-4	dihydroxyl +dehydrogen +glucuronid	12.00	$C_{17}H_{19}N_2O_9$	395.1075	395.1091	219.0773, 201.0668	+	-	+	-	-	-
M25-1	dihydroxyl +glucuronid	2.64	$C_{17}H_{21}N_2O_9$	397.1249	397.1247	221.0934, 203.0871, 160.0565	+	-	-	-	-	-
M25-2	dihydroxyl +glucuronid	4.91	$C_{17}H_{21}N_2O_9$	397.1231	397.1247	221.0922, 203.0820, 185.0711, 161.0726	+	-	-	-	-	-
M26-1	dihydroxyl +methyl +glucuronid	19.97	$C_{18}H_{23}N_2O_9$	411.1402	411.1404	187.0852	+	-	-	-	-	-
M26-2	dihydroxyl +methyl +glucuronid	20.40	$C_{18}H_{23}N_2O_9$	411.1356	411.1404	187.0873	+	-	-	-	-	-
M26-3	dihydroxyl +methyl +glucuronid	21.30	$C_{18}H_{23}N_2O_9$	411.1397	411.1404	187.0876	+	-	-	-	-	-
M26-4	dihydroxyl +methyl +glucuronid	21.93	$C_{18}H_{23}N_2O_9$	411.1402	411.1404	187.0856	+	-	-	-	-	-
M26-5	dihydroxyl +methyl +glucuronid	22.52	$C_{18}H_{23}N_2O_9$	411.1365	411.1404	187.0905	+	-	-	-	-	-
M26-6	dihydroxyl +methyl +glucuronid	22.79	$C_{18}H_{23}N_2O_9$	411.1359	411.1404	187.0861	+	-	-	-	-	-
M26-7	dihydroxyl +methyl +glucuronid	22.99	$C_{18}H_{23}N_2O_9$	411.1398	411.1404	187.0893	+	-	-	-	-	-
M26-8	dihydroxyl +methyl +glucuronid	23.48	$C_{18}H_{23}N_2O_9$	411.1379	411.1404	187.0884	+	-	-	-	-	-
M27	trihydroxyl +glucuronid	2.89	$C_{17}H_{21}N_2O_{10}$	413.1178	413.1196	237.0902, 187.0793, 160.0645	+	-	-	-	-	-
M28	acetyl+methyl +glucuronid	11.51	$C_{20}H_{25}N_2O_8$	421.1597	421.1611	245.1295, 187.0869, 169.0782, 144.0701	+	+	+	+	-	-
M29	hydroxyl+acetyl +methyl +glucuronid	7.61	$C_{20}H_{25}N_2O_9$	437.1583	437.1560	261.1233	+	-	-	-	-	-
M30	hydroxylation					+diglucuronidation						
2.97			$C_{23}H_{29}N_2O_{14}$	557.1573	557.1619	381.1342, 205.0970, 187.0867	+	-	-	-	-	-

Hydroxyl: hydroxylation; dehydrogen: dehydrogenation; methyl: methylation; monohydroxyl: monohydroxylation; dihydroxyl: dihydroxylation; didehydrogen: didehydrogenation; trihydroxyl: trihydroxylation; acetyl: acetylation; methoxyl: methoxylation; sulf: sulfation; glucuronid: glucuronidation; diglucuronid: diglucuronidation; U: urine; F: feces; B: bile; P: plasma; L: rat liver microsomes; H: rat primary hepatocytes.

doi:10.1371/journal.pone.0122366.t003

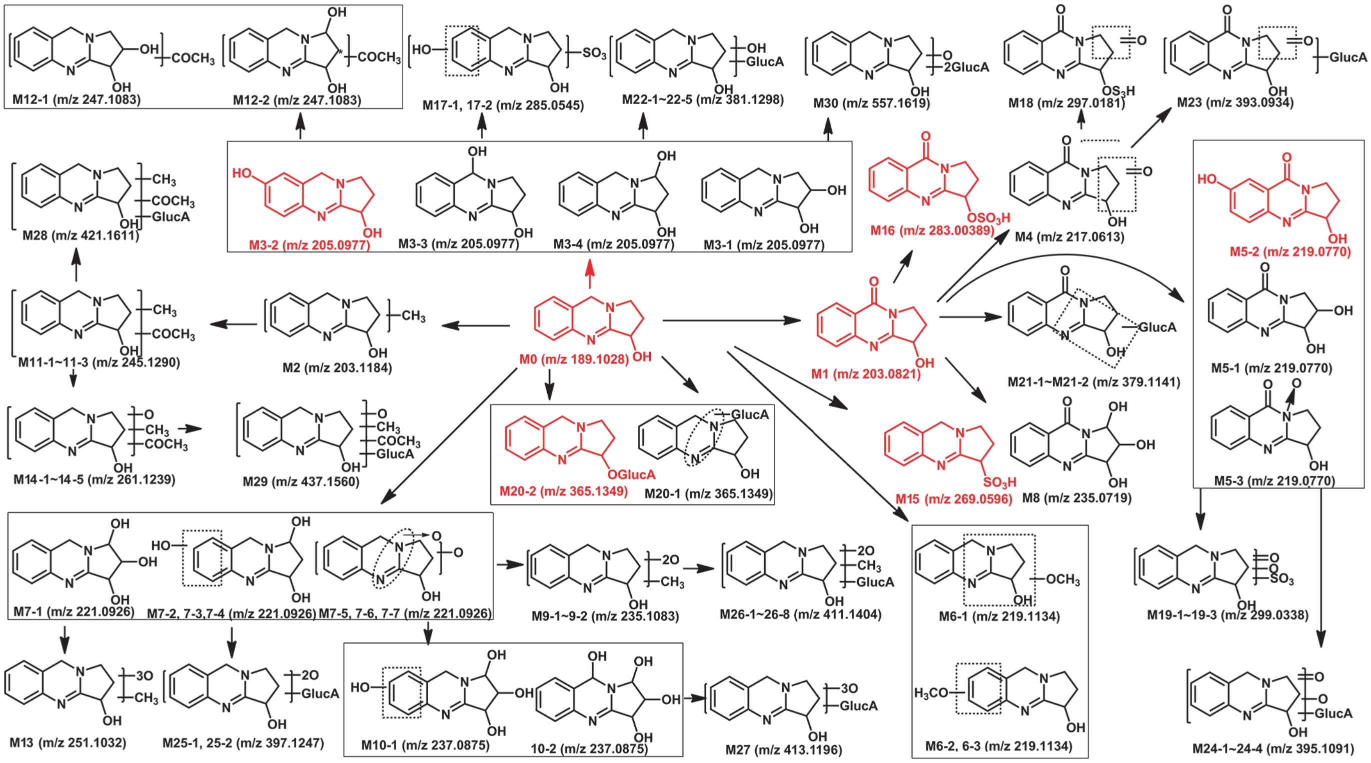

Fig 9. Proposed metabolic pathways of VAS.

doi:10.1371/journal.pone.0122366.g009

Metabolite M3-2 was detected at 2.81 min. The MS^2 spectra showed fragment ions at *m/z* 187.0882 ($C_{11}H_{11}N_2O^+$, calculated *m/z* 187.0871), 185.0730 ($C_{11}H_9N_2O^+$, calculated *m/z* 185.0715), 169.0775 ($C_{11}H_9N_2^+$, calculated *m/z* 169.0766), 160.0755 ($C_{10}H_{10}NO^+$, calculated *m/z* 160.0762), 159.0683 ($C_{10}H_9NO^+$, calculated *m/z* 159.0684), and 134.0618 ($C_8H_8NO^+$, calculated *m/z* 134.0606). The fragment ions at *m/z* 159.0683 and 134.0618 were attributed to the hydroxylation of ring A, suggesting that the +16 Da modification occurred on the aromatic moiety. The molecular formula of the metabolite was further supported by the ^1H and ^{13}C NMR spectral data (Table 4). The ^1H-NMR spectra of M3-2 in CD_3OD-d4 indicated the existence of three aromatic protons at δ 7.04 (1H, d, J = 8.7 Hz, H-5), 6.83 (1H, dd, J = 8.7, 2.5 Hz, H-6) and 6.69 (1H, d, J = 2.4 Hz, H-8), respectively. Compared with the parent compound, the spectra revealed that metabolic substitution occurred in the benzene ring (position C-6 or C-7). Meanwhile, the ^1H and ^{13}C NMR spectra of M3-2 was similar to that of VASL [29, 30]. Thus, M3-2 was confirmed as VASL.

Metabolite M3-4 was detected at 3.79 min. The MS2 spectra showed fragment ions at *m/z* 187.0879 ($C_{11}H_{11}N_2O^+$, calculated *m/z* 187.0871), 169.0775 ($C_{11}H_9N_2^+$, calculated *m/z* 169.0766), 161.0715 ($C_9H_9NO^+$, calculated *m/z* 161.0715), 133.0760 ($C_{10}H_9NO^+$, calculated *m/z* 133.0766), and 106.0675 ($C_8H_8NO^+$, calculated *m/z* 106.0657). The key ion *m/z* 133.0766 was generated from the ring C cleavage. Another key ion *m/z* 161.0715 was generated by loss of H_2O and C_2H_2 unit; the ion was 28 Da heavier than 133.0766. Our data indicated that hydroxylation occurred on ring C (position C-1). As a result, M3-4 was confirmed as 1,2,3,9-tetrahydropyrrolo[2,1-b]quinazolin-1,3-diol.

Metabolites M3-1 and M3-3 were detected at 2.58 and 3.53 min, respectively. The MS^2 spectra showed fragment ions at *m/z* 187.0875 ($C_{11}H_{11}N_2O^+$, calculated *m/z* 187.0871), 169.0751

Table 4. ^{1}H- and ^{13}C-NMR signals of metabolites M1, M3-2, M5-2, M15, M16, M20-2 and parent compound M0 (ppm).

NO.	Carbon signals							Proton signals						
	M0	M1	M3-2	M5-2	M15	M16	M20-2	M0	M1	M3-2	M5-2	M15	M16	M20-2
1	47.2	43.5	47.2	44.7	46.3	45.8	46.3	3.25 (1H, m)	3.87 (1H, m)	3.77 (1H, m)	4.05 (1H, m)	3.53 (1H, m)	4.01 (1H, m)	3.48[a] (1H, m)
								3.37 (1H, m)	4.09 (1H, m)	3.68 (1H, m)	4.29 (1H, m)	3.66 (1H, m)	4.23 (1H, m)	3.48[a] (1H, m)
2	29.8	29.9	30.2	30.6	27.2	28.5	27.8	1.92 (1H, m)	2.00 (1H, m)	2.14 (1H, m)	2.19 (1H, m)	2.46 (1H, m)	2.45 (1H, m)	2.30 (1H, m)
								2.35 (1H, m)	2.45 (1H, m)	2.69 (1H, m)	2.70 (1H, m)	2.62 (1H, m)	2.75 (1H, m)	2.66 (1H, m)
3	72.1	71.7	71.8	72.5	74.8	78.4	77.6	4.55 (1H, m)	4.97 (1H, m)	5.17 (1H, m)	5.20 (1H, t, J = 7.2 Hz)	5.45 (1H, m)	5.73 (1H, m)	3.62 (1H, m)
3a	164.3	160.4	162.4	158.6	160.5	158.1	156.0	-	-	-	-	-	-	-
5	120.4	120.9	119.7	125.6	117.8	120.5	116.6	6.97[a] (1H, m)	7.69 (1H, m)	7.04 (1H, d, J = 8.7 Hz)	7.44 (1H, dd, J = 8.9, 2.9 Hz)	7.07[a] (1H,m)	7.58 (1H, d, J = 8.2 Hz)	6.97[a] (1H, m)
6	125.8	134.7	116.9	122.2	126.4	136.8	127.0	7.12 (1H, m)	7.80 (1H, m)	6.83 (1H, dd, J = 8.7, 2.5 Hz)	7.56 (1H, d, J = 2.8 Hz)	7.24 (1H,m)	7.79 (1H, t, J = 7.8 Hz)	7.12 (1H, m)
7	123.6	126.8	156.6	156.7	119.1	127.1	117.2	6.97[a] (1H, m)	7.50 (1H, m)	-	-	7.13 (1H,m)	7.52 (1H, t, J = 7.6 Hz)	6.97[a] (1H, m)
8	127.4	127.4	114.4	110.0	127.0	129.4	127.4	6.92 (1H, d, J = 7.3 Hz)	8.12 (1H, m)	6.69 (1H, d, J = 2.4 Hz)	7.70 (1H, d, J = 8.8 Hz)	7.07[a] (1H, m)	8.03 (1H, d, J = 8.0 Hz)	6.92 (1H, d, J = 7.3 Hz)
9	49.1	160.9	51.5	162.9	50.5	162.4	51.3	4.57 (2H, s)	-	-	-	4.75 (2H, m)	-	3.71[a] (2H, m)
8a	129.5	126.1	119.4	129.3	129.1	125.9	129.3	-						-
4a	142.2	149.4	124.3	143.5	143.4	146.4	130.2	-						-
1'							101.9							5.26 (1H, t, J = 7.6 Hz)
2'-5'							75.4, 72.4, 71.5, 76.1							-
6'							175.0							

a, overlapping signals

doi:10.1371/journal.pone.0122366.t004

($C_{11}H_9N_29^+$, calculated *m/z* 169.0766), 159.0922 ($C_{10}H_{11}N_2^+$, calculated *m/z* 159.0922), and 130.0651 ($C_9H_8N^+$, calculated *m/z* 130.0657). The fragment ions at *m/z* 169.0751 and 159.0922 had successive loss of two H_2O molecules (36 Da) and another molecule of H_2O and a neutral loss of CO unit, respectively. Our data indicated that hydroxylation occurred on ring B or ring C (position C-2, C-9). LogP value, an octanol/water partition coefficient, was used to determine the site of hydroxylation. As an important parameter for estimating the hydrophobicity of a compound, LogP also be used to describe the tendency for distribution from aqueous phase into hydrophobic phase. Compounds with a high LogP would have long retention time on reverse phase high performance liquid chromatography [31, 32]. Considering that 1,2,3,9-tetrahydropyrrolo [2,1-b] quinazolin-2,3-diol had a smaller LogP value (0.45, calculated by ChemBioDraw Ultra 12.0) than 1,2,3,9-tetrahydropyrrolo [2,1-b] quinazolin-3,9-diol (LogP = 1.02), M3-1 could be determined as 1,2,3,9-tetrahydropyrrolo [2,1-b] quinazolin-2,3-diol and M3-3 as 1,2,3,9-tetrahydropyrrolo [2,1-b] quinazolin-3,9-diol.

Metabolite M4. Metabolite M4 was detected at 10.56 min. Metabolite M4 exhibited a $[M + H]^+$ ion at *m/z* 217.0606, which was 28 Da (27.9578 Da) higher than that of the protonated parent compound, indicating the introduction of two oxygen atom with didehydrogenation (+ 27.9585 Da). The metabolism site may be located in C-1, C-2, or C-9 of the parent compound. The MS^2 spectra showed identical fragment ions at *m/z* 199.0500 ($C_{11}H_7N_2O_2^+$, calculated *m/z* 190.0508), 189.0654 ($C_{10}H_9N_2O_2^+$, calculated *m/z* 189.0651), 160.0390 ($C_9H_8NO_2^+$, calculated *m/z* 160.0399), 147.0561 ($C_8H_7N_2O^+$, calculated *m/z* 147.0558), and 144.0464 ($C_9H_6NO^+$, calculated *m/z* 144.0449). The fragment ion at *m/z* 147.0561 was attributed to an oxygen atom with a dehydrogenated ring B (C-9). However, the position of the other dehydrogenated oxygen atom was unclear.

Metabolites M5. M5 was found to have a protonated molecular weight of 219.0770, which was 30 Da (29.9742 Da) higher than that of the protonated parent compound, indicating the introduction of dihydroxylation and dehydrogenation metabolite. In addition, three independent chromatographic peaks with protonated ions at *m/z* 219.0770 were detected.

M5-2 was detected at 11.27 min. The MS^2 spectra revealed fragment ions at *m/z* 201.0675 ($C_{11}H_9N_2O_2^+$, calculated *m/z* 201.0664), 183.0568 ($C_{11}H_7N_2O^+$, calculated *m/z* 183.0558), 173.0724 ($C_{10}H_9N_2O^+$, calculated *m/z* 173.0715), and 146.0589 ($C_9H_8NO^+$, calculated *m/z* 146.0606). The fragment ion at *m/z* 146.0589 was attributed to the hydroxylation of ring A. The ^1H- and ^{13}C-NMR data are presented in Table 4. The ^1H-NMR spectra of M5-2 in CD_3OD-d4 indicated the existence of three aromatic protons at δ 7.44 (1H, dd, J = 8.9, 2.9 Hz, H-5), 7.56 (1H, d, J = 2.8 Hz, H-6), and 7.70 (1H, d, J = 8.8 Hz, H-8). The spectra revealed that hydroxyl substitution occurred in the benzene ring. The carbonyl signals at δ162.9 in the ^{13}C-NMR spectra confirmed that M5-2 had a carbonyl group. The ^1H and ^{13}C NMR spectra of M5-2 were similar to that of VAOL. As a result, M5-2 was identified as VAOL.

M5-1 was detected at 10.01 min. The MS^2 spectra showed fragment ions at *m/z* 201.0652 ($C_{11}H_9N_2O_2^+$, calculated *m/z* 201.0664), 183.0562 ($C_{11}H_7N_2O^+$, calculated *m/z* 183.0558), 173.0703 ($C_{10}H_9N_2O^+$, calculated *m/z* 173.0715), 160.0674 ($C_9H_8N_2O^+$, calculated *m/z* 160.0637), and 132.0448 ($C_8H_6NO^+$, calculated *m/z* 132.0449). The fragment ions at *m/z* 160.0674 and 132.0448 were attributed to an oxygen atom with dehydrogenated ring B (C-9). Our data indicated that another hydroxylation occurred on ring C (position C-1 or C-2). Thus, M5-1 was identified as 1,3-dihydroxy-2,3-dihydropyrrolo [2,1-b] quinazolin-9 (1H)-one or 2,3-dihydroxy-2,3-dihydropyrrolo [2,1-b] quinazolin-9 (1H)-one. The LogP values of 1,3-dihydroxy-2,3-dihydropyrrolo [2,1-b] quinazolin-9 (1H)-one and 2,3-dihydroxy-2,3-dihydropyrrolo [2,1-b] quinazolin-9 (1H)-one were 0.62 and −0.12, respectively. Given that M5-2 had longer retention time of 11.27 min than that of M5-1 (10.01 min) and the LogP value of M5-2

(VAOL) was 0.14, M5-1 was identified as 2,3-dihydroxy-2,3-dihydropyrrolo [2,1-b] quinazolin-9 (1H)-one.

Metabolite M5-3 was detected at 16.18 min. The MS^2 spectra showed fragment ions at *m/z* 201.0675 ($C_{11}H_9N_2O_2^+$, calculated *m/z* 201.0664), 173.0728 ($C_{10}H_9N_2O^+$, calculated *m/z* 173.0715), 155.0625 ($C_{10}H_7N_2^+$, calculated *m/z* 155.0609), and 130.0668 ($C_9H_9N^+$, calculated *m/z* 130.0657). The key ion of *m/z* 173.0728 was generated by the loss of CO at ring B, which indicated that an oxygen atom underwent dehydrogenation at ring B (C-9). Meanwhile, the key ion of *m/z* 155.0608 and 130.0674 were generated by loss of H_2O and a molecule from the CHN unit. These data were attributed to an oxygen atom at ring C (position C-1 or position N). The chromatographic retention time of M5-3 (16.18 min) was longer than that of the precursor compound M1 (VAO) under the acidic elution condition (0.1% formic acid water-methanol), indicating that M5-3 was an *N*-oxide of VAO. Thus, M5-3 was identified as 3-hydroxy-9-oxo-2, 3, 9, 10-tetrahydro-1H-pyrrolo[2,1-b] quinazoline 10-oxide.

Metabolites M6. M6 was detected with a protonated molecular weight of 219.1134, which was 30 Da (30.0106 Da, CH_2O) higher than that of the protonated parent compound. These metabolites had a common special fragment ion 187.0825 and lost a molecule of methanol (CH_3OH), indicating the introduction of the methoxylation metabolite. In addition, three independent chromatographic peaks with protonated ions at *m/z* 219.1134 were detected.

M6-1 was detected at 2.10 min. The MS^2 spectra showed fragment ions at *m/z* 187.0825 ($C_{11}H_{11}N_2O^+$, calculated *m/z* 187.0871), 171.0934 ($C_{11}H_{11}N_2^+$, calculated *m/z* 171.0922), 158.0621 ($C_{10}H_8NO^+$, calculated *m/z* 158.0606), 132.0438 ($C_8H_6NO^+$, calculated *m/z* 132.0449), and 130.0676 ($C_9H_8N^+$, calculated *m/z* 130.0657). The fragment ions at *m/z* 158.0621, 132.0438, and 130.0676 were attributed to the methoxy group of ring B or ring C.

M6-2 and M6-3 were detected at 3.46 and 4.04 min, respectively. The MS^2 spectra showed fragment ions at *m/z* 187.0875 ($C_{11}H_{11}N_2O^+$, calculated *m/z* 187.0871), 171.0935 ($C_{11}H_{11}N_2^+$, calculated *m/z* 171.0922), 169.0781 ($C_{11}H_9N_2^+$, calculated *m/z* 109.0766), 160.0648 ($C_9H_8N_2O^+$, calculated *m/z* 160.0637), 159.0581 ($C_9H_7N_2O^+$, calculated *m/z* 159.0558), and 131.0618 ($C_8H_7N_2^+$, calculated *m/z* 131.0609). The fragment ions at *m/z* 160.0648 and 131.0618 were attributed to the methoxy group of ring A.

Metabolites M7. M7 was detected with a protonated molecular weight of 221.0926, which was 32 Da (31.9898 Da) higher than that of the protonated parent compound, indicating the introduction of dihydroxylation metabolites. In addition, seven independent chromatographic peaks with protonated ions at *m/z* 221.0926 were detected.

M7-1 was detected at 2.06 min. The MS^2 spectra showed fragment ions at *m/z* 203.0825 ($C_{11}H_{11}N_2O_2^+$, calculated *m/z* 203.0821), 175.0896 ($C_{10}H_{11}N_2O^+$, calculated *m/z*175.0871), 158.0601 ($C_{10}H_8NO^+$, calculated *m/z* 158.0601), 157.0771 ($C_{10}H_9N_2^+$, calculated *m/z*157.0766), and 130.0669 ($C_9H_8N^+$, calculated *m/z* 130.0657). The fragment ions at *m/z* 203.0848, 175.0836, 146.0845, and 130.0696 were generated by loss of molecule of H_2O; molecule of H_2O and CO unit; molecule of H_2O, CO and CHO unit; and molecule of H_2O, CO and CH_3NO unit, respectively. These findings were attributed the hydroxylation of ring C (positon C-1 and position C-2). As a result, M7-1 was identified as 1, 2, 3, 9-tetrahydropyrrolo [2,1-b] quinazoline-1,2,3-triol.

M7-2, M7-3, and M7-4 were detected at 2.62, 3.60, and 4.10 min, respectively. The MS^2 spectra showed fragment ions at *m/z* 203.0820 ($C_{11}H_{11}N_2O_2^+$, calculated *m/z* 203.0821), 185.0700 ($C_{11}H_9N_2O^+$, calculated *m/z* 185.0715), 161.0710($C_9H_9N_2O^+$, calculated *m/z* 161.0715), and 133.0767 ($C_8H_9N_2^+$, calculated *m/z* 133.0766). The key ion at *m/z* 161.0715 and 133.0767 were generated by loss of molecule of H_2O and C_2H_2O unit, and molecule of H_2O, C_2H_2O and CO unit. These findings were attributed to the hydroxylation of ring C (positon C-1). However, the position of the other hydroxyl group was unclear.

M7-5, M7-6, and M7-7 were detected at 5.81, 10.66, and 13.57 min, respectively. These retention times were longer than that of the parent compound and all given information of monohydroxylation metabolites. Thus, M7-5, M7-6, and M7-7 were identified as the *N*-oxides after hydroxylation or di- *N*-oxides of the parent compound.

M7-5 was detected at 5.81 min. The MS^2 spectra showed fragment ions at *m/z* 203.0848 ($C_{11}H_{11}N_2O_2^+$, calculated *m/z* 203.0821), 185.0754 ($C_{11}H_9N_2O^+$, calculated *m/z* 185.0715), 175.0836 ($C_{10}H_{11}N_2O^+$, calculated *m/z*175.0871), 146.0845 ($C_9H_{10}N_2^+$, calculated *m/z* 146.0844), and 130.0696 ($C_9H_8N^+$, calculated *m/z* 130.0657). The fragment ions at *m/z* 203.0848, 175.0836, 146.0845, and 130.0696 were generated by loss of a water molecule; molecule of H_2O and CO unit; molecule of H_2O, CO and CHO unit; and molecule of H_2O, CO and CH_3NO unit, respectively. However, the data obtained were not enough to identify the position of *N*-oxidation and other oxygen atoms.

M7-6 was detected at 10.66 min. The MS^2 spectra showed fragment ions at *m/z* 203.0859 ($C_{11}H_{11}N_2O_2^+$, calculated *m/z* 203.0821), 185.0735 ($C_{11}H_9N_2O^+$, calculated *m/z* 185.0715), 173.0730 ($C_{10}H_9N_2O^+$, calculated *m/z* 173.0715), 160.0419 ($C_9H_8NO_2^+$, calculated *m/z* 160.0399), and 132.0435 ($C_8H_6NO^+$, calculated *m/z* 132.0449). The fragment ions at *m/z* 160.0419 and 132.0435 were generated by the loss of H_2O molecule and C_2H_5N unit and a molecule of H_2O, C_2H_5N, and CO unit. However, our data were not enough to identify the position of *N*-oxidation and other oxygen atoms.

M7-7 was detected at 13.57 min. The MS^2 spectra showed fragment ions at *m/z* 203.0830 ($C_{11}H_{11}N_2O_2^+$, calculated *m/z* 203.0821), 185.0702 ($C_{11}H_9N_2O^+$, calculated *m/z* 185.0715), 173.0709 ($C_{10}H_9N_2O^+$, calculated *m/z* 173.0715), 155.0608 ($C_{10}H_7N_2^+$, calculated *m/z* 155.0609), and 130.0674 ($C_9H_8N^+$, calculated *m/z* 130.0657). The fragment ions at *m/z* 203.0830 and 173.0709 were generated by loss of H_2O and a molecule of H_2O and CH_2O unit, respectively. However, our data were not enough to identify the position of *N*-oxylation and other oxygen atoms.

Metabolite M8. M8 was detected at 14.94 min. M8 showed a $[M+H]^+$ ion at *m/z* 235.0727, which was 46 Da (45.9699 Da) higher than that of the protonated parent compound, indicating the introduction of three oxygen atom with dehydrogenation (+ 45.9691 Da). The MS^2 spectra showed identical fragment ions at *m/z* 217.0623 ($C_{11}H_9N_2O_3^+$, calculated *m/z* 217.061), 199.0516 ($C_{11}H_7N_2O_2^+$, calculated *m/z* 190.0508), 175.0722 ($C_9H_7N_2O_2^+$, calculated *m/z* 175.0708), 173.0519 ($C_{10}H_9N_2O^+$, calculated *m/z* 173.0515), and 147.0561 ($C_8H_7N_2O^+$, calculated *m/z* 147.0558). The fragment ion at *m/z* 147.0561 was attributed to an oxygen atom with dehydrogenated ring B (C-9). The fragment ions at *m/z* 217.0623 and 175.0722 were generated by loss of H_2O and a molecule of H_2O and C_2H_2O unit. Our data indicated hydrolation in ring C (position C-2). Meanwhile, the key ion at *m/z* 147.0567 was generated by the loss of H_2O, C_2H_2O, and CO unit, indicating that another hydroxyl group was combined in ring C (position C-3). Therefore, M8 was identified as 1,2,3-trihydroxy-2,3-dihydro-pyrrolo [2,1-b] quinazolin-9 (1H)-one.

Metabolites M9. M9 was detected with a protonated molecular weight of 235.1083, which was 46 Da (46.0057 Da) higher than that of the protonated parent compound, indicating the introduction of two oxygen atoms and a methyl group into the molecule. In addition, two independent chromatographic peaks with protonated ions at *m/z* 235.1083 were detected.

M9-1 and M9-2 were detected at 13.92 and 19.56 min, respectively. The MS^2 spectra were not acquired because of the interference of endogenous or other metabolites with same retention time and near molecular weight. Therefore, no fragment information could help to identify the structures of M9-1 and M9-2.

Metabolites M10. M10 was detected with a protonated molecular weight of 237.0875, which was 48 Da (47.9747 Da) higher than that of the protonated parent compound, indicating

the introduction of three oxygen atoms into the molecule. In addition, two independent chromatographic peaks with protonated ions at *m/z* 237.0875 were detected.

M10-1 was detected at 9.81 min. The MS^2 spectra showed fragment ions at *m/z* 219.0741 ($C_{11}H_{11}N_2O_3^+$, calculated *m/z* 219.0770), 201.0718 ($C_{11}H_9N_2O_2^+$, calculated *m/z* 201.0664), 189.0674 ($C_{10}H_9N_2O_2^+$, calculated *m/z* 189.0664), 160.0666 ($C_9H_8N_2O^+$, calculated *m/z* 160.0637), and 146.0618 ($C_9H_8NO^+$, calculated *m/z* 146.606). The fragment ion at *m/z* 146.0618 was attributed to the hydroxylation of ring A (position unclear). The fragment ions at *m/z* 189.0664 and 160.0666 were generated by loss of two H_2O molecules, a CHO unit, and a C_2H_2O unit. Our data were attributed to the hydroxylation of ring C (positon C-1 and C-2). Therefore, M10-1 was tentatively identified as 1,2,3,9-tetrahydropyrrolo [2,1-b] quinazoline-1,2,3,5 (6,7, or 8)-tetraol.

M10-2 was detected at 13.36 min. The MS^2 spectra showed fragment ions at *m/z* 219.0774 ($C_{11}H_{11}N_2O_3^+$, calculated *m/z* 219.0770), 201.0670 ($C_{11}H_9N_2O_2^+$, calculated *m/z* 201.0664), 183.0563 ($C_{11}H_7N_2O^+$, calculated *m/z* 183.0558), 159.0571 ($C_9H_7N_2O^+$, calculated *m/z* 159.0558), 157.0770 ($C_{10}H_9N_2^+$, calculated *m/z* 157.0766) and 132.0459 ($C_8H_6NO^+$, calculated *m/z* 132.0449). The fragment ion at *m/z* 132.0459 was attributed to the hydroxylation of ring B (position C-9). The fragment ions at *m/z* 201.0670, 159.0571, and 157.0770 were generated by the loss of two molecules of H_2O; two molecules of H_2O and C_2H_2O unit; two molecules of H_2O and CO_2 unit, respectively. Our findings were attributed to two hydroxylation of ring C (positions C-1 and C-2). Therefore, M10-2 was tentatively identified as 1,2,3,9-tetrahydropyrrolo [2, 1-b] quinazoline-1,2,3,9-tetraol.

Metabolites M11. M11 was detected with a protonated molecular weight of 245.1290, which was 56 Da (56.0262 Da) higher than that of the protonated parent compound, indicating the introduction of acetyl and methyl groups into the molecule. In addition, three independent chromatographic peaks with protonated ions at *m/z* 245.1290 were detected.

M11-1, M11-2, and M11-3 were detected at 8.05, 8.38, and 10.63 min, respectively. The MS^2 spectra showed fragment ions at *m/z* 203.1181 ($C_{11}H_{15}N_2O^+$, calculated *m/z* 203.1184), 187.0885 ($C_{11}H_{11}N_2O^+$, calculated *m/z* 187.0871), 169.0785 ($C_{11}H_9N_2^+$, calculated *m/z* 169.0766), 160.0647 ($C_9H_8N_2O^+$, calculated *m/z* 160.0637), 159.0602 ($C_9H_7N_2O^+$, calculated *m/z* 159.0558), and 131.0625 ($C_8H_7N_2^+$, calculated *m/z* 131.0609). The fragment ions at *m/z* 203.1181 (−42.0109 Da) and 187.0885 (−58.0405 Da) were attributed to acetylation and methylation of the parent compound. However, the concrete substitution position was unclear.

Metabolites M12. M12 was detected with a protonated molecular weight of 247.1083, which was 58 Da (58.0055 Da) higher than that of the protonated parent compound, indicating the introduction of oxygen atom and C_2H_2O unit (acetyl group) into the molecule. In addition, two independent chromatographic peaks with protonated ions at *m/z* 247.1083 were detected.

M12-1 and M12-2 were detected at 4.00 and 4.42 min, respectively. The MS^2 spectra showed fragment ions at *m/z* 229.0963 ($C_{13}H_{13}N_2O_2^+$, calculated *m/z* 229.0977), 187.0848 ($C_{11}H_{11}N_2O^+$, calculated *m/z* 187.0871), 169.0753 ($C_{11}H_9N_2^+$, calculated *m/z* 169.0766), 160.0770 ($C_{10}H_{10}NO^+$, calculated *m/z* 160.0762), and 131.0607 ($C_8H_7N_2^+$, calculated *m/z* 131.0609). The fragment ions of *m/z* 229.0963 (−18.0120 Da) and 187.0848 (−60.0235 Da) indicated a hydrogenation and acetylation occurring in parent compound. The fragment ion of *m/z* 131.0607 showed no oxygen atom substitution at rings B and A, but was attributed to the hydroxylation of ring C (position C-1 or C-2). However, the concrete position of acetyl group substitution was unclear.

Metabolite M13. M13 was detected (t_R = 13.47 min) with a protonated molecular weight of 251.1290, which was 62 Da (61.9997 Da) higher than that of the protonated parent compound, indicating the introduction of three oxygen atoms and a methyl group into the molecule. The MS^2 spectra showed fragment ions at *m/z* 219.0755 ($C_{11}H_{11}N_2O_3^+$, calculated *m/z*

219.0770), 201.0676 ($C_{11}H_9N_2O_2^+$, calculated *m/z* 201.0664), 183.0570 ($C_{11}H_7N_2O^+$, calculated *m/z* 183.0558), and 157.0786 ($C_{10}H_9N_2^+$, calculated *m/z* 157.0766). The fragment ion at *m/z* 219.0755 (−32.0270 Da) was generated by the loss of a methanol (CH_3OH) molecule. The fragment ions at *m/z* 201.0676 and 183.0570 (−58.0405 Da) were generated by the loss of H_2O and methanol and a molecule of two H_2O and CH_3OH. Our data indicated three oxygen atom substitutions in the parent compound. However, the concrete substitution position was unclear.

Metabolites M14. M14 was detected with a protonated molecular weight of 261.1239, which was 72 Da (72.0211 Da) higher than that of the protonated parent compound, indicating the introduction of oxygen atom, acetyl group, and methyl group into the molecule. In addition, five independent chromatographic peaks with protonated ions at *m/z* 261.1239 were detected.

M14-1 and M14-4 were detected at 2.65 and 8.38 min, respectively. The MS^2 spectra showed fragment ions at *m/z* 244.1195 ($C_{14}H_{16}N_2O_2^+$, calculated *m/z* 244.1212), 188.0939 ($C_{11}H_{12}N_2O^+$, calculated *m/z* 188.0950), 187.0882 ($C_{11}H_{11}N_2O^+$, calculated *m/z* 187.0871), 160.0641 ($C_9H_8N_2O^+$, calculated *m/z* 160.0637), 144.0700 ($C_9H_8N_2^+$, calculated *m/z* 144.0687), and 131.0616 ($C_8H_7N_2^+$, calculated *m/z* 131.0609), respectively. The fragment ion at *m/z* 244.1195 (−17.0050 Da) was generated by loss of a hydroxyl group from the parent compound. The fragment ion at *m/z* 188.0939 (−73.0306 Da) was generated by loss of a CH_2O and C_2H_3O unit. The fragment ions of *m/z* 187.0882, 160.0641, and 131.0616 were as same as that of M11, which indicated that hydroxylation, acetylation, and methylation occurred in the parent compound. However, the concrete substitution position was unclear.

M14-2 was detected at 4.48 min. The MS^2 spectra showed fragment ions at *m/z* 244.1176 ($C_{14}H_{16}N_2O_2^+$, calculated *m/z* 244.1212), 226.1070 ($C_{14}H_{14}N_2O^+$, calculated *m/z* 226.1106), 188.0937 ($C_{11}H_{12}N_2O^+$, calculated *m/z* 188.0950), 187.0865 ($C_{11}H_{11}N_2O^+$, calculated *m/z* 187.0871), 169.0765 ($C_{11}H_9N_2^+$, calculated *m/z* 169.0766) and 160.0647 ($C_9H_8N_2O^+$, calculated *m/z* 160.0637). The fragment ions at *m/z* 244.1195 (−17.0050 Da) and 226.1070 (35.0169 Da) were generated by the loss of an-OH unit and molecule of-OH and H_2O from the parent compound. The fragment ion at *m/z* 188.0939 (−73.0306 Da) was generated by the loss of OH and C_3H_4O. Our data indicated that hydroxylation, acetylation, and methylation had occurred in parent compound. However, the concrete substitution position was unclear.

M14-3 was detected at 7.89 min. The MS^2 spectra showed fragment ions at *m/z* 231.1183 ($C_{13}H_{15}N_2O_2^+$, calculated *m/z* 231.1134), 188.0937 ($C_{11}H_{12}N_2O^+$, calculated *m/z* 188.0950), 160.0647 ($C_9H_8N_2O^+$, calculated *m/z* 160.0637), 144.0690 ($C_9H_8N_2^+$, calculated *m/z* 169.0766) and 121.0668 ($C_8H_9O^+$, calculated *m/z* 121.0653). The fragment ion at *m/z* 231.1183 (−30.0056 Da) indicated the methylation of parent compound. The fragment ion at *m/z* 121.0668 indicated acetylation of ring A (position unclear). The fragment ion at *m/z* 188.0939 (−73.0306 Da) was generated by the loss of OH and C_3H_4O. Our data indicated that hydroxylation, acetylation, and methylation had occurred in parent compound.

M14-5 was obviously detected at 15.76 min. The MS^2 spectra were not acquired because of the interference with endogenous or other metabolites having the same retention time and near molecular weight. Therefore, no fragment information could help to identify the structure of M14-5.

Metabolites M15. M15 was detected (t_R = 8.53 min) with a protonated molecular weight of 269.0609, which was 80 Da (79.9581 Da) higher than that of the protonated parent compound, indicating the introduction of sulfonic group into the molecule. The MS^2 spectra showed fragment ions at *m/z* 189.1038 ($C_{11}H_{13}N_2O^+$, calculated *m/z* 189.1028), 171.0932 ($C_{11}H_{11}N_2O^+$, calculated *m/z* 171.0922), 154.0672 ($C_{11}H_8N^+$, calculated *m/z* 154.0657), 144.0813 ($C_{10}H_{10}N^+$, calculated *m/z* 144.0813), and 118.0668 ($C_8H_8N^+$, calculated *m/z* 118.0657). The fragment ion at *m/z* 189.1038 (−79.9571 Da) indicated M15 as a sulfate at *m/z*

189.1038 molecular. Other fragment ion information was as same as those of the parent compound, indicating that M15 was 1,2,3,9-tetrahydro- pyrrolo [2, 1-b] quinazolin-3-yl hydrogen sulfate.

The molecular formula of the metabolite was further supported by the ^{1}H and ^{13}C NMR spectral data (Table 4). The ^{1}H-NMR spectra of M15 indicated the existence of four aromatic protons and the metabolic substitution did not occur in the benzene ring. Meanwhile, the ^{13}C NMR spectra of M15 were similar to that of VAS (M0). Difference was observed between the ^{1}H-NMR spectra of M15 and VAS. Only one of the protons at δ 5.45 (H-3) shifted to downfield by 0.90 ppm; the other protons signals shifted to a downfield by 0.28–0.54 ppm, which indicated that metabolic substitution occurred in C-3. Therefore, M15 was confirmed as 1,2,3,9-tetrahydropyrrolo [2,1-b] quinazolin-3-yl hydrogen sulfate.

Metabolite M16. M16 was detected (t_R = 10.82 min) with a protonated molecular weight of 283.0389, which was 94 Da (93.9361 Da) higher than that of the protonated parent compound, indicating the introduction of oxygen with dehydrogenation and sulfonic group into the molecule. The MS^2 spectra showed fragment ions at *m/z* 203.0826 ($C_{11}H_{11}N_2O_2^+$, calculated *m/z* 203.0821), 185.0725 ($C_{11}H_9N_2O^+$, calculated *m/z* 185.0715), 167.0624 ($C_{11}H_7N_2^+$, calculated *m/z* 167.0616), 157.0767 ($C_{10}H_9N_2^+$, calculated *m/z* 157.0766), 140.0529 ($C_{10}H_6N^+$, calculated *m/z* 140.0500), and 130.0653 ($C_9H_8N^+$, calculated *m/z* 130.0557). The fragment ion at *m/z* 203.0826 (−79.9563 Da) indicated M15 was a sulfate at *m/z* 203.0826 molecule. The other fragment ions information were as same as that of VAO, indicating that M16 was 9-oxo-1,2,3,9-tetrahydropyrrolo [2,1-b] quinazolin-3-yl hydrogen sulfate.

The molecular formula of the metabolite was further supported by the ^{1}H and ^{13}C NMR spectral data (Table 4). Similar to M15, the ^{1}H-NMR spectra of M16 indicated the existence of four aromatic protons, indicating that metabolic substitution did not occur in the benzene ring. Meanwhile, the ^{13}C NMR spectra of M16 were similar to the VAO (M1). Differences were observed between the ^{1}H-NMR spectra of M16 and VAO. Only one of the protons at δ 5.73 (H-3) shifted to downfield by 0.76 ppm; the other protons signals shifted to downfield by 0.14–0.45 ppm. This finding indicated that the metabolic substitution was happened in the C-3. Therefore, M16 was confirmed as 9-oxo-1,2,3,9-tetrahydropyrrolo [2,1-b] quinazolin-3-yl hydrogen sulfate.

Metabolites M17. M17 was detected with a protonated molecular weight of 285.0545, which was 96 Da (95.9517 Da) higher than that of the protonated parent compound, indicating the introduction of oxygen atom and sulfonic group into the molecule. In addition, two independent chromatographic peaks with protonated ions at *m/z* 285.0545 were detected.

M17-1 and M17-2 were detected at 2.24 and 4.69 min, respectively. The MS^2 spectra showed fragment ions at *m/z* 205.0981 ($C_{11}H_{13}N_2O_2^+$, calculated *m/z* 205.0977), 187.0882 ($C_{11}H_{11}N_2O^+$, calculated *m/z* 187.0871), 160.0617 ($C_9H_8N_2O^+$, calculated *m/z* 160.0762), and 134.0618 ($C_8H_8NO^+$, calculated *m/z* 134.0606). The fragment ion at *m/z* 205.0981 (-79.9564 Da) indicated that M16 was a sulfate of *m/z* 205.0981 molecule. Other fragment ion information was as same as that of M3-2 (*m/z* 205 Da). Therefore, M17-1 and M17-2 might be sulfates of M3-2, but the same fragment ion information with M3-2 might be found all of the hydroxylation occurred on ring A. Thus, M17-1 and M17-2 were identified as the sulfates of the parent compound that underwent hydroxylation at ring A.

Metabolite M18. M18 was detected (t_R = 10.56 min) with a protonated molecular weight of 297.0185, which was 108 Da (107.9157 Da) higher than that of the protonated parent compound. The MS^2 spectra showed identical fragment ions at *m/z* 217.0618 ($C_{11}H_9N_2O_3^+$, calculated *m/z* 217.0613), 199.0539 ($C_{11}H_7N_2O_2^+$, calculated *m/z* 190.0508), 160.0400 ($C_9H_6NO^+$, calculated *m/z* 160.0399), and 147.0590 ($C_8H_7N_2O^+$, calculated *m/z* 147.0558). The fragment ion at *m/z* 217.0618 (−79.9567 Da) indicated that M18 was a sulfate of *m/z* 217.0618 molecular.

The other fragment ion information was as same as that of M7 (*m/z* 217 Da). Therefore, M18 was identified as 1 (or 2), 9-dioxo-1,2,3,9-tetrahydro- pyrrolo [2,1-b] quinazolin-3-yl hydrogen sulfate.

Metabolite M19. Metabolites M19-1, M19-2, and M19-3 were detected at 6.37, 7.22, and 8.25 min, respectively. M19 showed a $[M+H]^+$ ion at *m/z* 299.0338, which was 110 Da (109.9310 Da) higher than that of the protonated parent compound. The MS^2 spectra showed obvious fragment ion at *m/z* 219.0766 ($C_{11}H_{11}N_2O_3^+$, calculated *m/z* 219.0770). Given the interference with endogenous or other metabolites having the same retention time and near molecular weight, no more fragment ions information of M19 could be acquired. Therefore, M19 was tentatively identified metabolites by di-hydroxylation, dehydrogenation, and sulfation of parent compound.

Metabolites M20. M20 was detected with a protonated molecular weight of 365.1349, which was 176 Da (176.0221 Da) higher than that of the protonated parent compound, indicating the introduction of $C_6H_8O_6$ unit (glucuronidation) into the molecule. In addition, two independent chromatographic peaks with protonated ions at *m/z* 365.1349 were detected.

M20-1 and M20-2 were detected at 7.90 and 8.25 min, respectively. The MS^2 spectra showed fragment ions at *m/z* 189.1023 ($C_{11}H_{13}N_2O^+$, calculated *m/z* 189.1028), 171.0924 ($C_{11}H_{11}N_2O^+$, calculated *m/z* 171.0922), 154.0644 ($C_{11}H_8N^+$, calculated *m/z* 154.0657), 144.0838 ($C_{10}H_{10}N^+$, calculated *m/z* 144.0813), and 118.0649 ($C_8H_8N^+$, calculated *m/z* 118.0657). The fragment ion at *m/z* 189.1023 (−176.0226 Da) indicated that M20 was a glucuronide conjugate of the parent compound. Other fragment ion information of M20 was as same as that of parent compound. These results revealed that M20 was a tentatively identified glucuronide conjugate of VAS (position C-3, N-1 or N-2).

The molecular formula of M20-2 was further supported by the ^1H and ^{13}C NMR spectral data (Table 4). The ^1H-NMR spectra of M20-2 indicated the existence of four aromatic protons and the metabolic substitution did not occur in the benzene ring. The proton signals at δ 5.26 (1H, t, J = 7.6 Hz, H-1') in the^1H-NMR spectra and the carbonyl signals at δ 101.9, 75.4, 72.4, 71.5, 76.1, 175.0 in the ^{13}C-NMR spectra confirmed that this metabolite had a β-D-glucuronic acid. The ^{13}C-NMR spectra of M20-2 were similar to the spectra of VAS (M0), except for the carbonyl signals of the glucuronic residue, indicating that the parent nucleus of M20-2 was VAS (M0). The HMBC correlations between the carbonyl signal at δ 77.6 (C-2) and proton signal at δ5.03 (1H, t, J = 7.6 Hz, H-1') indicated that the glucuronic acid was linked to the C-3. Thus, M20-2 was confirmed as 1,2,3,9-tetrahydropyrrolo [2,1-b] quinazolin-3-β-D-glucuronide, while M20-1 was tentatively identified as glucuronide conjugate of the parent compound (position N-1 or N-2).

Metabolites M21. M21 was detected with a protonated molecular weight of 379.1141, which was 190 Da (190.0113 Da) higher than that of the protonated parent compound, indicating the introduction of oxygen atom, dehydrogenation, and addition of a $C_6H_8O_6$ unit (glucuronidation) into the molecule. In addition, two independent chromatographic peaks with protonated ions at *m/z* 379.1141 were detected.

M21-1 and M21-2 were detected at 7.90 and 8.81 min, respectively. The MS^2 spectra showed fragment ions at *m/z* 203.0836 ($C_{11}H_{11}N_2O_2^+$, calculated *m/z* 203.0821), 185.0724 ($C_{11}H_9N_2O^+$, calculated *m/z* 185.0715). The fragment ion at *m/z* 203.0836 (−176.0305 Da) indicated that M21 was a glucuronide conjugate of VAO. M21 was tentatively identified as the glucuronide conjugate of VAO (positions C-3, N-1, or N-2).

Metabolites M22. M22 was detected with a protonated molecular weight of 381.1298, which was 192 Da (192.0270 Da) higher than that of the protonated parent compound, indicating the introduction of oxygen atom and addition of a $C_6H_8O_6$ unit (glucuronidation) into the

molecule. In addition, five independent chromatographic peaks with protonated ions at *m/z* 381.1298 were detected.

M22-1, M22-2, M22-3, and M22-4 were detected at 0.89, 1.25, 1.75, and 3.24 min, respectively. The MS^2 spectra showed fragment ions at *m/z* 205.0983 ($C_{11}H_{13}N_2O_2^+$, calculated *m/z* 205.0977), 187.0876 ($C_{11}H_{11}N_2O^+$, calculated *m/z* 187.0871), 160.0770 ($C_{10}H_{10}NO^+$, calculated *m/z* 160.0762), and 134.0598 ($C_8H_8NO^+$, calculated *m/z* 134.0606). The fragment ions at *m/z* 160.0770 and 134.0598 indicated the hydrogenation of ring A (position unclear). The fragment ion at *m/z* 205.0983 (−176.0315 Da) indicated that they were the products of the hydrogenation and glucuronidation of the parent compound.

M22-5 was detected at 3.84 min. The MS^2 spectra showed fragment ions at *m/z* 205.0976 ($C_{11}H_{13}N_2O_2^+$, calculated *m/z* 205.0977), 187.0876 ($C_{11}H_{11}N_2O^+$, calculated *m/z* 187.0871), 160.0425 ($C_9H_6NO_2^+$, calculated *m/z* 160.0399), and 136.0780 ($C_8H_{10}NO^+$, calculated *m/z* 136.0762). The fragment ion at *m/z* 160.0425 also indicated the hydrogenation of ring A (position unclear). The fragment ion at *m/z* 205.0976 (−176.0322 Da) might tentatively help to identify M22-5 as a product of hydrogenation and glucuronidation of the parent compound.

Metabolite M23. M23 was detected (t_R = 12.25 min) with a protonated molecular weight of 393.0943, which was 204 Da (203.9915 Da) higher than that of the protonated parent compound. The MS^2 spectra showed identical fragment ions at *m/z* 217.0611 ($C_{11}H_9N_2O_3^+$, calculated *m/z* 217.0613), 199.0539 ($C_{11}H_7N_2O_2^+$, calculated *m/z* 190.0508), 160.0600 ($C_9H_6NO^+$, calculated *m/z* 160.0399), and 147.0548 ($C_8H_7N_2O^+$, calculated *m/z* 147.0558). The fragment ion at *m/z* 217.0611 (−176.0332 Da) indicated that M23 was a glucuronide conjugate of *m/z* 217.0618 molecular. The other fragment ion information was as same as that of M7 (*m/z* 217 Da). Therefore, M23 was identified as a glucuronide conjugate of the parent compound by di-hydroxylation and di-dehydrogenation.

Metabolites M24. M24 was detected with a protonated molecular weight of 395.1091, which was 206 Da (206.0063 Da) higher than that of the protonated parent compound, indicating the introduction of two oxygen atom, dehydrogenation, and addition of a $C_6H_8O_6$ unit (glucuronidation) into the molecule. In addition, four independent chromatographic peaks with protonated ions at *m/z* 395.1091 were detected.

M24-1, M24-2, M24-3, and M24-4 were detected at 1.91, 4.92, 10.40, and 12.00 min, respectively. The MS^2 spectra showed fragment ions at *m/z* 219.0778 ($C_{11}H_{11}N_2O_3^+$, calculated *m/z* 219.0770), and 201.0659 ($C_{11}H_9N_2O_2^+$, calculated *m/z* 201.0664). The fragment ion at *m/z* 219.0778 (−176.0313 Da) indicated that M24 was a glucuronide conjugate by di-hydroxylation, dehydrogention of the parent compound. However, no more fragment information was acquired because the peak abundance of M24 was too low or the interference was too serious. Therefore, the concrete substitution position was unclear.

Metabolites M25. M25 was detected with a protonated molecular weight of 397.1247, which was 208 Da (208.0219 Da) higher than that of the protonated parent compound, indicating the introduction of two oxygen atom and addition of a $C_6H_8O_6$ unit (glucuronidation) into the molecule. In addition, two independent chromatographic peaks with protonated ions at *m/z* 397.1247 were detected.

M25-1 and M25-2 were detected at 2.64 and 4.91 min, respectively. The MS^2 spectra showed fragment ions at *m/z* 221.0934 ($C_{11}H_{13}N_2O_3^+$, calculated *m/z* 221.0926), and 203.0871 ($C_{11}H_{11}N_2O_2^+$, calculated *m/z* 203.0821). The fragment ion at *m/z* 221.0934 (−176.0313 Da) indicated that the M25 could be tentatively identified as glucuronide conjugate of parent compound after di-hydroxylation. However, the concrete substitution position was unclear.

Metabolites M26. M26 was detected with a protonated molecular weight of 411.1404, which was 222 Da (222.0376 Da) higher than that of the protonated parent compound, indicating the introduction of CH_2O_2 (methyl group and two oxygen atom) and addition of a $C_6H_8O_6$

unit (glucuronidation) into the molecule. In addition, eight independent chromatographic peaks with protonated ions at *m/z* 411.1404 were detected.

M26-1, M26-2, M26-3, M26-4, M26-5, M26-6, M26-7, and M26-8 were detected at 19.97, 20.46, 21.30, 21.93, 22.52, 22.79, 22.99, and 23.48 min, respectively. The MS^2 spectra showed fragment ion at *m/z* 187.0852 ($C_{11}H_{11}N_2O^+$, calculated *m/z* 187.0871). Given the interference with endogenous or other metabolites having same retention time and near molecular weight, no more fragment information could be obtained to identify the structure of metabolites. M26 was tentatively identified as the glucuronide conjugate of the parent compound after di-hydroxylation and methylation.

Metabolite M27. M27 was detected (t_R = 23.48 min) with a protonated molecular weight of 413.1196, which was 204 Da (203.9915 Da) higher than that of the protonated parent compound. The MS^2 spectra showed identical fragment ions at *m/z* 237.0902 ($C_{11}H_{13}N_2O_3^+$, calculated *m/z* 237.0875), 187.0793 ($C_{10}H_9N_2O_2^+$, calculated *m/z* 187.0871), and 160.0645 ($C_9H_8N_2O^+$, calculated *m/z* 160.0637). The fragment ion at *m/z* 237.0902 (−176.0294 Da) indicated M27 was a glucuronide conjugate of *m/z* 237.0902 molecule. M27 was identified as glucuronide conjugate of parent compound after tri-hydroxylation

Metabolite M28. M28 was detected (t_R = 11.51 min) with a protonated molecular weight of 421.1591, which was 232 Da (232.0563 Da) higher than that of the protonated parent compound. The MS^2 spectra showed identical fragment ions at *m/z* 245.1295 ($C_{14}H_{17}N_2O_2^+$, calculated *m/z* 245.1290), 187.0869 ($C_{10}H_9N_2O_2^+$, calculated *m/z* 187.0871), 169.0782 ($C_{11}H_9N_2^+$, calculated *m/z* 169.0766) and 144.0701 ($C_9H_8N_2^+$, calculated *m/z* 144.0687). The fragment ion at *m/z* 245.1295 (−176.0304 Da) indicated M28 was a glucuronide conjugate of *m/z* 245.1295 molecular. M28 was identified as glucuronide conjugate of parent compound after methylation and acetylation.

Metabolite M29. M29 was detected (t_R = 7.61 min) with a protonated molecular weight of 437.1583, which was 248 Da (248.0555 Da) higher than that of the protonated parent compound. The MS^2 spectra showed fragment ion at *m/z* 261.1233 ($C_{14}H_{17}N_2O_3^+$, calculated *m/z* 261.1239). The fragment ion at *m/z* 261.1233 (−176.0350 Da) indicated that M29 was a glucuronide conjugate of *m/z* 261.1233 molecule. Given that no more fragment ion information could be obtained to identify the structure of this metabolite, M29 was tentatively identified as the glucuronide conjugate of the parent compound after hydroxylation, methylation, and acetylation.

Metabolite M30. M30 was detected (t_R = 2.97 min) with a protonated molecular weight of 557.1573, which was 368 Da (368.0545 Da) higher than that of the protonated parent compound. The MS^2 spectra showed fragment ions at *m/z* 381.1342 ($C_{17}H_{21}N_2O_8^+$, calculated *m/z* 381.1298) and 205.0970 ($C_{11}H_{13}N_2O_2^+$, calculated *m/z* 205.0977). The fragment ions at *m/z* 381.1342 (−176.0232 Da) and 205.0970 (−352.0603) indicated that M30 was di-glucuronide conjugate of *m/z* 205.0970 molecule. M30 was then tentatively identified as di-glucuronide conjugate of parent compound after hydroxylation.

Anticholinesterase activity of VAS and metabolites

The inhibitory activities of VAS and its key metabolites of VAO, VASL, VAOL, VAS-3-S, VAO-3-S, and VAS-3-G against AChE and BChE were evaluated in vitro and summarized in Table 5. All tested compounds exhibited some degree of inhibitory activities on both AChE and BChE. The parent compound VAS exhibited strong inhibition both on AChE and BChE with IC_{50} values of 3.380 ± 0.030 and 0.101 ± 0.003 μM, respectively. As phase I metabolites of VAS, VASL also showed strong inhibition against both AChE and BChE activities with IC_{50} values of 2.649 ± 0.432 and 2.463 ± 0.224 μM, respectively. However, VAO exhibited low

Table 5. Inhibitory activity (IC_{50}) and selectivity index (SI) of the metabolites investigated against AChE and BChE.

Metabolites	IC_{50} (μM ± SD)		SI
	AChE	BChE	
M0 (VAS)	3.380 ± 0.030	0.101 ± 0.003	0.030
M1 (VAO)	76.597 ± 8.458	10.200 ± 2.083	0.133
M3-2 (VASL)	2.649 ± 0.432	2.463 ± 0.224	0.930
M5-2 (VAOL)	46.890 ± 2.835	72.450 ± 8.634	1.545
M15 (VAS-3-S)	49.600 ± 2.774	9.821 ± 0.925	0.198
M16 (VAO-3-S)	395.000 ± 19.545	27.290 ± 5.028	0.069
M20-2 (VAS-3-G)	20.770 ± 0.329	103.800 ± 15.522	4.998

IC_{50} values were determined by regression analyses and expressed as the means ± SD of three replicate determinations. The SI represents the AChE selectivity index defined as IC_{50} BChE/IC_{50} AChE.

doi:10.1371/journal.pone.0122366.t005

inhibitory activity against AChE, with IC_{50} values of 76.597 ± 8.458 μM, and moderate inhibitory activity against BChE, with IC_{50} values of 10.200 ± 2.083 μM. VAOL exhibited weaker inhibitory activity both on AChE and BChE activities with IC_{50} values of 46.890 ± 2.835 μM and 72.450 ± 8.634 μM, respectively. As the phase II metabolites of VAS, VAS-3-S exhibited weaker inhibitory activity against AChE, with IC_{50} values of 49.600 ± 2.774 μM, and slightly stronger inhibitory activity against BChE with IC_{50} values of 9.821 ± 0.925 μM. VAO-3-S exhibited low inhibitory activity against AChE with IC_{50} values of 395.000 ± 19.545 μM and moderate inhibitory activity against BChE with IC_{50} values of 27.290 ± 5.028 μM. VAS-3-G exhibited moderate inhibitory activity against AChE with IC_{50} values of 20.770 ± 0.329 μM and low inhibitory activity against BChE with IC_{50} values of 103.800 ± 15.522 μM.

Discussion

According to results of bioavailability studies of VAS and metabolites in our laboratory (no published data), the absolute bioavailability of VAS after oral administration was about 50%, and the elimination half-life of VAS after intravenous and oral administration was about 5 h and 1.5 h, respectively. In the process of urine samples collection, 15 rats have been administrated with VAS orally at dose of 100 mg/kg body weight once a day for 21 consecutive days and no any observable side effects have been seen. In addition, the LD_{50} value of VAS has been determined as 308.25 mg/kg (no published data). It illustrated that VAS is safe as a potential candidate drug.

In the present study, we systematically investigated the metabolites of VAS in vivo (rat urine, feces, plasma, and bile) and in vitro (RLMs and RPHs) and the cholinesterase inhibitory activities of its main metabolites. By using the analysis of UPLC/ESI-QTOF MS scan, ^{1}H-NMR, and ^{13}C NMR, a total of 72 metabolites were identified. Among them, 72 metabolites were found from the urine, 15 from the feces, 25 from the plasma, 45 from the bile, 18 from RLMs, and 11 from RPHs. These results indicated that VAS encountered a strong liver first pass effect and the liver was the momentous metabolic organ for the metabolism of VAS.

VAS undergoes extensive sequential metabolism in vivo and in vitro. Both the phases I and II metabolites were observed. The metabolic pathways of VAS were proposed in Fig 9. The formation of the phase I metabolites included hydroxylation (M3, M7, M10) and desaturation (M1, M4, M5, M8). Among the phase I metabolites, the product of monohydroxylation and desaturation (VAO) was the main metabolite. This compound could be separated from *P. harmala* and *A. vasica*. Other hydroxylation or hydroxylation and desaturation products were also

extensively observed. The reaction involved the addition of one, two, or even three hydroxyl groups or/and desaturation from the parent drug. In some metabolites, two metabolic transformations simultaneously occurred. For example, M8 was formed by the desaturation reaction followed by a hydroxylation reaction. This metabolic pattern is widespread in many cases [20]. The phase II biotransformation observed as methylation, acetylation, sulfation, and glucuronidation. Among the reactions, sulfation and glucuronidation metabolites (M15, M16, M20-2 and M21-2) were most common among phase II metabolites. To obtain the reference substances of VAS metabolites, 15 rats were given continuous oral administration of VAS for 21 days and urine samples were collected within a period of 0 h to 528 h (48 h after the last dosing) for chemical separation. M15, M16, M20-2, and M21-2 were then isolated from the urine. However, because of an unavoidable lapsus, most of M20-2 and M21-2 were hydrolyzed due to urine sample being exposed at room temperature for an excessively long time at a high pH environment ($pH > 10$). Only M15, M16, and a small quantity M20-2 were isolated and acquired. The structures were determined as VAS-3-S, VAO-3-S, and VAS-3-G by NMR and UPLC/ESI-QTOF-MS. It indicated that when we needed to isolate the metabolites from urine and feces, the pH value was a vital factor that must be controlled. The structure of metabolites indicated the 3-hydroxyl group and C-9 site are the main metabolic soft spots, and VAO was an important intermediate metabolite.

In a previous study, VAO, DVAS, DVAO, VAS-3-G, and VAO-3-G have been identified as metabolites of VAS in rats after a single oral dose of 20 mg/kg has been reported [16]. In our study, however, DVAS, DVAO, and their related metabolites could not be found either in vivo or in vitro. We believed that several factors could have caused the discrepancies. Firstly, VAS with impurity might be used in previous studies. We all know that the natural compounds isolated from plants could be easily mixed with some analogs. We found that the initial separation and preparation of VAS easily being resulted in some impurities (including DVAS and DVAO), when the parent compound VAS being isolated from *P. harmala*. In order to obtain pure VAS (not contain DVAS and DVAO), purification should be performed further. Secondly, the animal strain or individual differences could be a source of discrepancy. The metabolism of many drugs was also affected by the species, strain, sex, age, and individual differences [33]. Thirdly, the stereochemical structure of VAS would be given consideration for the discrepancy results. In addition, (-)-VAS was also isolated from *A. visica* and (±)-VAS was isolated from *P. harmala* [34]. The different of stereoselective characteristics might lead to different metabolism profiles [35].

For the 23 metabolites found in plasma, VAO, 1,2,3,9-tetrahydropyrrolo [2,1-b] quinazolin-3,9-diol (a hydroxylation metabolite of VAS, but the substituted site was not as same as VASL), VAS-3-S, VAO-3-S, VAS-3-G, and VAO-3-G were found with high abundance. VAO was reported to cause more bronchodilatory effects than parent VAS both in vitro and in vivo [36]. In addition, VAO was reported to have more potent anti-asthmatic activity than VAS comparable to that of sodium cromoglycate [37]. Meanwhile, as another important metabolic pathway, the sulfation metabolites were first reported for VAS and related compounds.

From the anti-cholinesterase assay results (Table 5), we can see that the parent compound VAS exhibited strong inhibition both on AChE and BChE activities, and that the tested metabolites exhibited inhibitory activity on both AChE and BChE, especially in the IC_{50} values of VASL (2.649 ± 0.432 μM) against AChE. It was slightly stronger than the parent compound (VAS, 3.242 ± 0.079 μM). Interestingly, the IC_{50} values of VAOL (46.890 ± 2.835 μM) against AChE was also stronger than VAO (76.597 ± 8.458 μM), which implied that the hydroxy-substituted benzene ring of VAS analogs could increase the inhibitory activity on AChE. However, the AChE selectivity index (SI, IC_{50} of BChE/IC_{50} of AChE) of VASL and VAOL were 0.930 and 1.545, which indicated that the hydroxy-substituted on benzene ring of VAS analogs

obviously could reduce the selectivity for BChE inhibition. In comparison with VAS and VAS-3-S, the inhibitory activities of VAO and VAO-3-S on AChE and BChE were significantly reduced. VAO and VAO-3-S were reduced by 22.7-fold and 8.0-fold on AChE inhibitory activity and 101.0-fold and 2.8-fold on BChE inhibitory activity, respectively. However, the AChE SI of VAO and VAO-3-S were unchanged compared with VAS and VAS-3-S, which implied that the ketonization of VAS analogs might cause reduced inhibitory activity both on AChE and BChE. In addition, to be compared with VAS, the activities of VAS-3-G against AChE and BChE have reduced 6.7-fold and 1027.7-fold, respectively, with reduced selectivity for BChE inhibition. Overall, VAS has undergone inactivation in vivo in respect to anticholinesterase activity.

In conclusion, our study revealed that VAS is a metabolic unstable compound that is extensively metabolized in vivo and in vitro in rat. The 3-hydroxyl group and C-9 site are the main metabolic soft spots, and VAO is an important metabolic intermediate. The result of anticholinesterase activity assay of VAS and its main metabolites indicated that VAS undergoes metabolic inactivation process in vivo in consideration of anticholinesterase activity. Results from our study are important in understanding the metabolism and anticholinesterase activity of VAS and related quinazoline type alkaloid and provide useful information that can be used as reference for pharmacochemistry and clinical pharmacology.

Acknowledgments

We thank *Ph.D* Yingbo Yang and *M.S* Haijun Hu for their help with the isolated and purification of metabolites from urine. And we also thank *Prof*. Guixin Chou for help with NMR instrumentation for identification of the structure of metabolites.

Author Contributions

Conceived and designed the experiments: CHW WL. Performed the experiments: WL XYS YDY XMC BHY. Analyzed the data: WL BJ XYS. Contributed reagents/materials/analysis tools: QL HH CYH YLW. Wrote the paper: CHW WL ZTW.

References

1. Liu W, Cheng XM, Wang ZT, Wang CH. Vasicine: research advances in resources, pharmacological activities,pharmacokinetics, toxicity and analysis methods. J Int Pharm Res. 2013; 40: 386–395.
2. Rachana, Sujata B, Mamta P, Priyanka KM, Sonam S. Review and future perspectives of using vasicine, and related compounds. Indo Global J Pharm Sci. 2011; 1: 85–98.
3. Wen FF, Zheng LM, Li XJ, Li Y, Zhang L, Cheng XM. Research on quality standards of herbs of *Peganum harmala*. China J Chinese Mater Med. 2012; 37: 2971–2976.
4. Ahmad S, Garg M, Ali M, Singh M, Athar MT, Ansari SH. A phytopharmacological overview on *Adhatoda zeylanica* Medic. syn. *A. vasica* (Linn.) Nees. Nat Prod Radi. 2009; 8: 549–554
5. Cheng XM, Zhao T, Yang T, Wang CH, Bligh S, Wang ZT. HPLC fingerprints combined with principal component analysis, hierarchical cluster analysis and linear discriminant analysis for the classification and differentiation of Peganum sp. indigenous to China. Phytochem Analysis. 2010; 21: 279–289. doi: 10.1002/pca.1198 PMID: 20020435
6. Zhao T, Ding KM, Zhang L, Cheng XM, Wang CH, Wang ZT. Acetylcholinesterase and butyrylcholinesterase inhibitory activities of β-carboline and quinoline alkaloids derivatives from the plants of genus Peganum. J Chem. 2013; doi: 10.1155/2013/71723
7. Zheng XY, Zhang L, Cheng XM, Zhang ZJ, Wang CH, Wang ZT. Identification of acetylcholinesterase inhibitors from seeds of plants of genus Peganum by thin-layer chromatography-bioautography. JPC J Planar Chromat. 2011; 24: 470–474.
8. Liu W, Yang YD, Cheng XM, Gong C, Li SP, He DD, et al. Rapid and sensitive detection of the inhibitive activities of acetyl- and butyryl-cholinesterases inhibitors by UPLC-ESI-MS/MS. J Pharm Biomed Anal. 2014; 94: 215–220 doi: 10.1016/j.jpba.2014.02.004 PMID: 24631841

9. Singh B, Sharma RA. Anti-inflammatory and antimicrobial properties of pyrroloquinazoline alkaloids from *Adhatoda vasica* Nees. Phytomedicine. 2013; 20: 441–445. doi: 10.1016/j.phymed.2012.12.015 PMID: 23357363

10. Srinivasarao D, Jayaraj I, Jayaraj R, Prabha ML. A study on antioxidant and anti-inflammatory activity of vasicine against lung damage in rats. Indian J Allergy Asthma. 2006; 20: 1–7.

11. Mahindroo N, Ahmed Z, Bhagat A, Lal Bedi K, Kant Khajuria R, Kapoor VK, et al. Synthesis and structure-activity relationships of vasicine analogues as bronchodilatory agents. Med Chem Res. 2005; 14: 347–368.

12. Chattopadhyay N, Nosál'ová G, Saha S, Bandyopadhyay SS, Flešková D, Ray B. Structural features and antitussive activity of water extracted polysaccharide from *Adhatoda vasica*. Carbohydrate Polymers. 2011; 83: 1970–1974.

13. Nepali K, Sharma S, Ojha R, Dhar KL. Vasicine and structurally related quinazolines. Med Chem Res. 2013; 22: 1–15

14. Lin JH, Lu AY. Role of pharmacokinetics and metabolism in drug discovery and development. Pharmacol Rev. 1997; 49: 403–449. PMID: 9443165

15. Trunzer M, Faller B, Zimmerlin A. Metabolic soft spot identification and compound optimization in early discovery phases using MetaSite and LC-MS/MS validation. J Med Chem. 2009; 52: 329–335 doi: 10.1021/jm8008663 PMID: 19108654

16. Sharma SC, Siddiqi MA, Zutshi U, Atal CK. The *in vivo* metabolism of vasicine-a potent uterotonic. Indian Drugs. 1983; 20: 431–434.

17. Holčapek M, Kolářová L, Nobilis M. High-performance liquid chromatography-tandem mass spectrometry in the identification and determination of phase I and phase II drug metabolites. Anal Bioanal Chem. 2008; 391: 59–78. doi: 10.1007/s00216-008-1962-7 PMID: 18345532

18. Levsen K, Schiebel HM, Behnke B, Dötzer R, Dreher W, Elend M, et al. Structure elucidation of phase II metabolites by tandem mass spectrometry: an overview. J Chromatogr A. 2005; 1067: 55–72. PMID: 15844510

19. Bateman KP, Castro-Perez J, Wrona M, Shockcor JP, Yu K, Oballa R, et al. MS^E with mass defect filtering for *in vitro* and *in vivo* metabolite identification. Rapid Commun Mass Spectrom. 2007; 21: 1485–1496. PMID: 17394128

20. Ibáñez M, Sancho JV, Pozo OJ, Hernández F. Use of quadrupole time-of-flight mass spectrometry to determine proposed structures of transformation products of the herbicide bromacil after water chlorination. Rapid Commun Mass Spectrom. 2011; 25: 3103–3113. doi: 10.1002/rcm.5183 PMID: 21953966

21. Zhang H, Zhang D, Ray K, Zhu M. Mass defect filter technique and its applications to drug metabolite identification by high-resolution mass spectrometry. J Mass Spectrom. 2009; 44: 999–1016. doi: 10.1002/jms.1610 PMID: 19598168

22. Cheng CR, Yang M, Yu K, Guan SH, Tao SJ, Millar A, et al. Identification of metabolites of ganoderic acid D by ultra-performance liquid chromatography/quadrupole time-of-flight mass spectrometry. Drug Metab Dispos. 2012; 40: 2307–2314. doi: 10.1124/dmd.112.047506 PMID: 22942320

23. Li L, Chen XY, Li D, Zhong DF. Identification of 20(S)-protopanaxadiol metabolites in human liver microsomes and human hepatocytes. Drug Metab Dispos. 2011; 39: 472–483. doi: 10.1124/dmd.110.036723 PMID: 21139039

24. Zheng XY, Zhang ZJ, Chou GX, Wu T, Cheng XM, Wang CH, et al. Acetylcholinesterase inhibitive activity-guided isolation of two new alkaloids from seeds of *Peganum nigellastrum* Bunge by an in vitro TLC- bioautographic assay. Arch Pharm Res. 2009; 32: 1245–1251. doi: 10.1007/s12272-009-1910-x PMID: 19784581

25. Walsky RL, Obach RS. Validated assays for human cytochrome P450 activities. Drug Metab Dispos. 2004; 32: 647–660. PMID: 15155557

26. Lowry OH, Rosebrough NJ, Farr AL, Randall RJ. Protein measurement with the Folin phenol reagent. J Biol Chem. 1951; 193: 265–275. PMID: 14907713

27. Schrenk D, Karger A, Lipp HP, Bock KW. 2, 3, 7, 8-Tetrachlorodibenzo-p-dioxin and ethinylestradiol as co-mitogens in cultured rat hepatocytes. Carcinogenesis. 1992; 13: 453–456. PMID: 1547536

28. Rashkes YV, Karimov A, Telezhenetskaya MV, Yunusov SY. A comparison of the mass spectra of isomeric hydroxy and methoxy analogs of deoxypeganine and deoxyvasicinone. Chem Nat Compd+. 1983; 19: 64–68.

29. Gao H, Huang YN, Gao B, Li P, Inagaki C, Kawabata J. Inhibitory effect on α-glucosidase by *Adhatoda vasica* Nees. Food Chem. 2008; 108: 965–972.

30. Hua H, Cheng M, Li X, Pei Y. A new pyrroloquinazoline alkaloid from *Linaria vulgaris*. Chem Pharm Bul. 2002; 50: 1393–1394.

31. Hanai T, Hubert J. Prediction of retention time of phenols in liquid chromatography. J High Res Chrom. 1983; 6: 20–26.

32. Li SP, Liu W, Teng L, Cheng XM, Wang ZT, Wang CH. Metabolites identification of harmane in vitro/in vivo in rats by ultra-performance liquid chromatography combined with electrospray ionization quadrupole time-of-flight tandem mass spectrometry. J Pharm Biomed Anal. 2014; 92: 53–62 doi: 10.1016/j.jpba.2014.01.003 PMID: 24486683

33. Lin G, Cui YY, Liu XQ, Wang ZT. Species differences in the *in vitro* metabolic activation of the hepatotoxic pyrrolizidine alkaloid clivorine. Chem Res Toxicol. 2002; 15: 1421–1428. PMID: 12437333

34. Joshi BS, Newton MG, Lee DW, Barber AD, Pelletier SW. Reversal of absolute stereochemistry of the pyrrolo[2,1-b]quinazoline alkaloids vasicine, vasicinone, vasicinol and vasicinolone. Tetrahedron: Asymmetry. 1996; 7: 25–28.

35. Niwa T, Shiraga T, Mitani Y, Terakawa M, Tokuma Y, Kagayama A. Stereoselective metabolism of cibenzoline, an antiarrhythmic drug, by human and rat liver microsomes: possible involvement of CYP2D and CYP3A. Drug Metab Dispos. 2000; 28: 1128–1134. PMID: 10950860

36. Swamy G, Ravikumar K. Crystal and molecular structures of quinazolines: A ring conformational study. J Chem Crystallogr. 2005; 35: 183–189.

37. Bhide M, Naik P, Ghooi R. Comparative studies on the pharmacological evaluation of vasicine and vasicinone. Bull Haffkin Inst. 1976; 4: 45–50.
